# Voting suffrage and the political budget cycle: Evidence from the London Metropolitan Boroughs 1902–1937

**DOI:** 10.1016/j.jpubeco.2014.01.003

**Published:** 2014-04

**Authors:** Toke S. Aidt, Graham Mooney

**Affiliations:** a Faculty of Economics, Jesus College, University of Cambridge, Cambridge CB3 9DD, United Kingdom; b Institute of the History of Medicine, Johns Hopkins University, 1900 East Monument St., Baltimore, MD 21205, USA

**Keywords:** Local public finance, Voting franchise, Suffrage, Opportunistic political budget cycles, London

## Abstract

We study the opportunistic political budget cycle in the London Metropolitan Boroughs between 1902 and 1937 under two different suffrage regimes: taxpayer suffrage (1902–1914) and universal suffrage (1921–1937). We argue and find supporting evidence that the political budget cycle operates differently under the two types of suffrage. Taxpayer suffrage, where the right to vote and the obligation to pay local taxes are linked, encourages demands for retrenchment and the political budget cycle manifests itself in election year tax cuts and savings on administration costs. Universal suffrage, where all adult residents can vote irrespective of their taxpayer status, creates demands for productive public services and the political budget cycle manifests itself in election year hikes in capital spending and a reduction in current spending.

## Highlights

• Comparative examination of the political budget cycle under different suffrage rules.
• Study of the conditional political budget cycle for the pre-WWII period in London.
• Taxpayer suffrage encourages election year tax cuts and savings on administration.
• Universal suffrage encourages election year expenditure switching.

## 1 Introduction

Suffrage rules regulate who can vote and this, in turn, influences the interests served by elected politicians. While today we associate democracy with equal and universal suffrage, historically the power to elect or appoint representatives was the privilege of narrow elites. Suffrage rules focussed on specific characteristics of the individual such as ownership of property, payment of taxes, residency and gender. The logic behind linking the right to vote to property holdings or tax payments can be traced back to mediaeval Britain and reflected the belief that it restricted the franchise to individuals with a longer-term interest in the welfare of the community, akin to the shareholders of corporations.

Economic models in the tradition of Meltzer and Richard (1981) predict a straightforward positive link between demands for public goods and redistribution and extension of the franchise. However, evidence from analyses of historical data show that the impacts were more complex than predicted by theory and were functions of the specific rules that determined who could vote.^2^ While progress has been made in understanding the public finance consequences of franchise extension, little is known about the influence these rules have on the incentive to manipulate tax and spending patterns prior to elections in the quest for votes. A well-established literature, drawing on evidence from modern democracies and surveyed by Paldam (1997), Alesina et al. (1997) and most recently by Drazen (2008), offers a strong argument for the existence of opportunistic political budget cycles in both national and local elections.^3^ The construction of cross-country datasets (of OECD countries and more recently of developing countries) and of rich datasets for local governments (municipalities or states) from the modern period has tended to draw attention to the experience of the late 20th and early 21st centuries at the expense of earlier periods. Consequently the focus has been on opportunistic political budget cycles operating under universal suffrage; quite how the cycle might manifest itself in polities with economic and social restrictions on who could vote has been completely overlooked.

The purpose of this paper is to draw upon the historical experience of early 20th century London to study the nature of the political budget cycle under two different suffrage regimes: taxpayer suffrage, where the right to vote is linked to specific tax payments; and universal suffrage, where all adults can vote (with minor qualifications), irrespective of their economic status. While the identity of the “pivotal voter” differs systematically under the two suffrage rules, electorally-motivated politicians can be expected to be equally determined to manipulate fiscal policy before elections to win support from the pivotal voter. We, therefore, conjecture that an opportunistic political budget cycle will be present in both regimes but that its nature will vary systematically with the suffrage rules.^4^

The setting for our study is the London Metropolitan Boroughs (LMBs) before and after the First World War. The 28 LMBs were established in 1899 and had powers to levy local property taxes, to decide on the provision of local services (sewer connections, bathhouses, parks, libraries, dairies and milk shops, etc.) and to take out loans to finance capital expenses on the security of future property taxes. Within the statutory boundaries, the LMBs had significant fiscal autonomy and the elected representatives of the councils could decide on the level, composition and the timing of key fiscal variables. All councillors were elected every three years. The franchise before the First World War was based on property tax payment and restricted to men; we refer to it as taxpayer suffrage. The Representation of the People Act (sometimes referred to as the Fourth Reform Act) in 1918 eradicated the tax payment requirement at all levels of government (including for the LMBs) and introduced almost equal and universal suffrage.^5^ This quasi-natural experiment allows us to study the opportunistic political budget cycle under two different suffrage regimes.^6^

Besides adding new historical evidence to the debate on the opportunistic political budget cycle, our study contributes directly to two more specific strands of literature.^7^ Firstly, it significantly enhances our understanding of fiscal retrenchment and taxpayer democracy in Britain. Until 1918, voting rights in local elections linked representation to the prompt payment of the local property tax (known in Britain as the rate) such that only local taxpayers had the right to vote. This had intriguing implications for the relationship between the size of the electorate and local public finance. In particular when the balance of power shifted to small-scale, middle class taxpayer-voters, demands were made for retrenchment and economy rather than fiscal expansion, despite apparently large social returns on public investment in local public goods (Hennock, 1963, 1973; Wohl, 1983; Szreter, 1988, 1997). As documented by Aidt et al. (2010), this generated a negative relationship between spending on local public goods and the extension of the franchise.^8^ We add to this by studying how opportunistic political budget cycles operate in an environment with taxpayer-voters. This restricted franchise is compared to the regime of universal suffrage, where the pivotal voter often does not contribute much to the local tax base.

Secondly, our study contributes to the fast expanding research on the *conditional* political budget cycle initiated by Persson and Tabellini (2003), Brender and Drazen (2005), Shi and Svensson (2006), Alt and Lassen (2006a,b) and Alt and Rose (2007) amongst others, and recently surveyed by de Haan and Klomp (2013). The general point here is that the size and nature of the political budget cycle are conditional on the political and economic environment. They depend, amongst other factors, on economic conditions (e.g., the level of income), the institutional framework (e.g., the level of corruption, the type of election or political system), and the monitoring framework (e.g., fiscal transparency and quality of the press). We add an important dimension to this conditionality by showing that the opportunistic political budget cycle is influenced by the details of the franchise.

We find the following results. Under taxpayer suffrage (1902–1914), the opportunistic political budget cycle materializes as tax cuts and in reduced spending on administration in election years. Under universal suffrage (1921–1937), we find that expenditures in election years are shifted towards productive public goods (capital spending) and away from other types of (current) spending, with no effect on tax income. The LMBs operated under a balanced budget rule which limited their ability to deficit finance election year tax cuts or spending booms, yet we find evidence of smaller surpluses in election years under both suffrage regimes.

We interpret these findings in the light of the different incentives that variations in the suffrage rules generate for politicians to engineer opportunistic cycles. Building on Lohmann (1998), Shi and Svensson (2006), and Aidt et al. (2010), we provide a formal rational choice model that illustrates the logic. Under a restricted taxpayer suffrage that explicitly disenfranchises non-taxpayers and enfranchises owners of property in the locality who can reside elsewhere, taxpayer-voters often demand retrenchment and economy. Politicians respond to this by cutting taxes and reducing spending on administration in election years, as we observe in the data. In contrast, under universal suffrage all adult residents hold the right to vote, including many poorer residents who contribute little in terms of property tax payments to the funding of spending. This generates demand for fiscal expansion. Politicians, therefore, aim to engineer additional electoral support by adjusting the portfolio of spending towards productive public services which benefit the pivotal voter and away from other spending without necessarily increasing taxes.

The rest of the paper is organized as follows. In Section 2, we introduce the institutional setting of our study and the particularities of the suffrage rules governing elections to the councils of the LMBs before and after the First World War. In Section 3, we develop the theoretical foundation for our empirical investigation. To this end, we sketch a rational choice model and provide an online supplementary appendix with technical details. In Section 4, we present the data and discuss some stylized facts about local public finance in London between 1902 and 1937. In Section 5, we consider the evidence of an opportunistic political budget cycle. In Section 6, we lay out our empirical strategy. We present the main findings in Section 7 and in Section 8 we discuss alternative interpretations and robustness checks. The concluding remarks in Section 9 recapitulate our findings in the context of conditional political budget cycles.

## 2 The institutional setting

The 28 LMBs were established by the London Government Act of 1899 and they took office in November 1900 (Robson, 1939, chapter 10; Young and Garside, 1982).^9^ LMBs were created from the largest of the existing Vestries and District Boards of Works and by combining smaller Vestries and Boards into bigger and fiscally more viable units.^10^ As with the Vestries and District Boards, the main responsibility of the boroughs was the provision of local urban amenities. This included construction and maintenance of local streets, refuse collection, provision of public lighting (by 1912, 15 LMBs were generating their own electricity for street lighting), sewers and drainage, burial grounds, libraries, parks, baths and washhouses, and the employment of health officers. They could also purchase land and build public sector housing (White, 2001). Other services, such as schools, infectious disease hospitals, policing and major roads and infrastructure projects fell outside their jurisdiction and were handled by a variety of city-wide authorities, but the bulk of spending on sanitation and health-related public services was undertaken by the boroughs.^11^ The responsibilities stayed constant over the period from 1901 to 1937 (in fact to the 1960s) and there were no substantial changes in fiscal federalism over the period.^12^

The main source of LMB revenue was receipts from the rate – the local property tax – which often contributed around 90% of total income. User charges for specific services were also important and some equalization funds were available, though poorer boroughs complained about the iniquity of the redistribution (Booth, 2009). From this base, the boroughs provided local public goods and financed the administrative cost of running the council. They also collected taxes on behalf of other local authorities (e.g., the School Board for London, London County Council, the Boards of Guardians, and the Metropolitan Police). Within these institutional and fiscal constraints, the elected councillors had freedom to allocate public monies as they saw fit and to raise the tax resources they deemed necessary to fund required expenditures. While they could borrow funds for the purpose of capital investment, they were not allowed to do so to finance current spending and they effectively operated under a balanced budget rule which, however, did not preclude surpluses.

The LMBs were governed by a council, consisting of a mayor, aldermen and councillors.^13^ These were elected in competitive elections. Unlike prior to 1901, where elections for the Vestries took place each year for a third of the vestrymen, all the LMBs adopted an election cycle in which the entire council was elected every three years.^14^ The rules governing the electoral franchise for the LMBs between 1901 and 1918 were codified in the Local Government Act of 1894. Voters consisted of two groups of men (and a limited number of widows and spinsters): the Parochial Electors and the Parliamentary Electors were entitled to vote under the Parliamentary Reform Act of 1884 and the Registration Act of 1885 (Keith-Lucas, 1952, p. 233). Both groups were required to occupy a property in the borough for a sufficient time period (ranging from 6 to 12 months), but permanent residence in the borough was not necessary. Some boroughs, therefore, had a significant number of absentee voters. Most importantly, however, eligibility to vote for the council was linked directly to payment of the rate. Provided that the occupancy requirement was satisfied, the right to vote was conferred on occupiers of property worth at least £10 and had been subject to 12 months' rating with the rate paid in full. This implied that the right to vote was restricted to the taxpayers of the borough who had paid their dues on time and in full. This disenfranchised many poorer inhabitants. Since the fraction of the total stock of property rated in each borough varied (slum areas were sometimes not rated) as did the diligence of tax collection, the fraction of males aged 20 and above that could vote varied greatly. In 1909, for example, about 37% of adult males in Stepney and 78% of adult males in Battersea were eligible.^15^ The average extension of the franchise across the boroughs between 1902 and 1914 was about 60%. We refer to this as the taxpayer suffrage.

Taxpayer suffrage was abolished by the Representation of the People Act of 1918 which established one standard franchise for all general and local elections in Great Britain. For men the requirement was six months' occupation of land or premises in the area (i.e., no tax payment requirement). The condition for women was six months' occupation of land or premises in the area or as the wife of a man so qualified, on account of premises in which they both resided, if she was 30 years old (Keith-Lucas, 1952, p. 235). The Act also abolished the disenfranchisement of paupers for all local government purposes. While owners of land or buildings within the borough previously were entitled to vote whether they lived in the borough or not, after 1918 they qualified to be elected as a borough councillor, but not to vote. Although some women had to wait until 1928 to get the right to vote, we refer to this post-1918 situation as universal suffrage.

## 3 The budget cycle and the suffrage: a theoretical framework

We consider a borough populated by capitalists (C) and workers (L) during two periods, *t* = 1,2. Each capitalist is endowed with capital (*k*) and two units of housing. Workers are endowed with one unit of labour, which is supplied in-elastically to a competitive labour market, and nothing else. There are *n_c_* capitalists and more workers than that. Each period, the capitalists combine their capital endowment with hired labour to produce output using a CRTS technology. The market clearing wage and profit income, *w*_*t*_^⁎^ and *π*_*t*_^⁎^, are both strictly increasing in total factor productivity. The capitalists “consume” one unit of housing privately and pay the property tax levied on it directly. The other unit is supplied to a competitive market as rental accommodation for workers. Under the assumption that the supply of houses is fixed, the incidence of the property tax levied on rented accommodation, if any, falls on the capitalists and workers therefore do not pay the local property tax.

An elected council determines the borough's fiscal affairs. An election takes place between the two periods. We assume that the council is run by a capitalist-politician who is rewarded with an exogenous per-period ego-rent *M* and endogenous rents, *r_t_*, extracted by diverting tax revenues to private income with *r_t_* ≤ *r*^⁎^. The tasks of the capitalist-politician are to provide productive and non-productive public goods and to raise the funds needed through property taxation. The productive public good, *g_t_*, makes the borough economy more productive and wages and profits are strictly increasing and concave functions of *g_t_*. The non-productive public good, *q_t_*, only benefits capitalists (property owners) and is a normal good. All this is financed by the local property tax, *τ_t_*, which is levied on each house. The budget constraint is

(1) $$\tau_{t}=\frac{r_{t}+q_{t}+g_{t}}{2n_{c}},$$

where the tax base is 2*n_c_* because each capitalist owns two units of housing. Residents' wellbeing depends on three factors: the budget allocation, the quality of the politician running the council, and random events (luck). The utility generated by the budget allocation (and consumption of private goods) is

(2) $$v_{C}\left(q_{t},g_{t},r_{t}\right)\equiv u_{C}\left(p,q_{t},\pi^{*}\left(g_{t}\right)-\frac{r_{t}+g_{t}+q_{t}}{n_{C}}\right)$$

(3) $$v_{L}\left(g_{t}\right)\equiv u_{L}\left(p,w^{*}\left(g_{t}\right)\right)$$

where *u_C_* and *u_L_* are standard indirect utility functions defined over the price of the private good (p), the non-productive public good (for capitalists) and income, which in the case of capitalists is net of the property tax needed to balance the budget. All residents benefit from the productive public good because it increases wage and profit income. Workers want as much of this good provided as possible. Taxpaying capitalists face a trade-off between the higher profits earned in a more productive economy and the utility they get from the non-productive good and the cost of paying the necessary taxes. They view rents as waste and want this cut to zero. The per-period utility of a capitalist-politician is

(4) $$S_{C}\left(q_{t},g_{t},r_{t}\right)\equiv u_{C}\left(p,q_{t},\pi^{*}\left(g_{t}\right)-\frac{r_{t}+g_{t}+q_{t}}{n_{C}}+r_{t}\right)$$

which we notice is increasing in the rent. While all capitalist-politicians share this objective function and care about re-election, they differ with regard to “quality”. Quality matters for residents because the utility they get from a given budget allocation increases with the quality of the incumbent politician. The total utility of capitalists and workers is

(5) $$V_{t}^{C}=v_{C}\left(q_{t},g_{t},r_{t}\right)+\eta_{t}+\mu_{t}$$

(6) $$V_{t}^{L}=v_{L}\left(g_{t}\right)+\eta_{t}+\mu_{t},$$

where *η_t_* is the quality shock, which determines how competent the incumbent is, and *μ_t_* is a “luck” shock that may make him look more (or less) competent than may be the case.The fundamental information assumption of the model is that voters observe total utility but are unable to decompose this into the three sub-components before the election. The model captures the notion that voters, typically, are ill-informed about the finer details of local public finance; or if they do know, they cannot (except at equilibrium) say for sure if the welfare they derive from the budget policy is due to the policy itself or to other factors such as the quality of the politician or simply to luck.^16^ While the two shocks are unobserved, they are drawn from known normal distributions with zero mean and variance *σ*_*η*_^2^ and *σ*_*μ*_^2^, respectively. The “luck” shock is drawn independently each period. The competency shock is an attribute of a politician and, if the incumbent capitalist-politician is re-elected, then the competency shock from period 1 also applies to period 2. A new capitalist-politician elected for period 2 is associated with a new draw. This information structure introduces a moral hazard element which is the source of the rational political budget cycle. The suffrage rules determine who can vote in the election at the end of period 1. Under taxpayer suffrage (*TS*), only the owners of property, i.e., capitalists, can vote. In contrast, under universal suffrage (*US*), all residents can vote and the pivotal voter is a worker. In both suffrage regimes, the timing of events is:

1. At the beginning of period 1, a balanced budget {*g*_1_,*q*_1_,*r*_1_} is implemented by the incumbent capitalist-politician.
2. The two random shocks *η*_1_ and *μ*_1_ are realized but not observed directly by anyone.
3. Total utility is determined and observed by all residents.
4. At the end of the period, the election takes place. Those with the right to vote either re-elect the incumbent capitalist-politician or elect a “new” capitalist-politician.
5. The winner implements a balanced budget {*g*_2_,*q*_2_,*r*_2_} for period 2.
6. The “luck” shock, *μ*_2_, is realized. If the politician is “new”, the competency shock, *η*_2_, is realized. If the incumbent politician was re-elected, the competency shock from period 1 (*η*_1_) carries over to period 2.
7. Total utility is determined and observed by all residents.

In period 2, the capitalist-politician implements the balanced budget policy that maximizes *S*_*C*_(*q*_2_,*g*_2_,*r*_2_) subject to *r*_2_ ≤ r*.^17^ The optimal post-election budget is {g*,q*,r*} with $\tau^{*}=\frac{g^{*}+q^{*}+r^{*}}{2n_{C}}$. The level of productive public goods *g*^⁎^ maximizes profit income net of the tax cost for a representative capitalist but the level of the non-productive public good is higher than capitalist-voters want because the capitalist-politician enriches himself with the maximum rent (*r*^⁎^) and *q* is a normal good. In period 1, to improve his re-election prospects, the incumbent must increase the likelihood of appearing competent by delivering extra utility to the appropriate group of voters. We first find the “utility target” which the incumbent wants to “engineer” in the quest for re-election under each suffrage regime. Given these “utility targets”, we characterize the underlying pre-election budget policy. The derivation of the “utility targets” is formally similar to the analysis of Lohmann (1998). Intuitively, voters want to re-elect an incumbent of above average quality, but neither they nor the incumbent observes the quality shock *η*_1_ directly. Voters do, however, observe their total utility and they know the equilibrium budget choice of the incumbent. By solving the resulting signal extraction problem, they arrive at a Bayesian estimate of the incumbent's quality. They can, then, adopt a rational retrospective voting rule which re-elects the incumbent if and only if total utility is above a threshold. This, in turn, provides the incumbent with an incentive to engineer a pre-election increase in the utility of the pivotal voters in the knowledge that this will make him appear competent. We denote the resulting “utility targets” *U_TS_* and *U_US_* and note that *U*_*TS*_ > *v*_*C*_(g*,q*,r*) and *U*_*US*_ > *v*_*L*_(g*). Given the “utility targets”, the equilibrium pre-election budget maximizes *S*_*C*_(*g*_1_,*q*_1_,*r*_1_) subject to *r*_1_ ≤ r* and to the relevant re-election constraint. Under taxpayer suffrage, the re-election constraint is *v*_*C*_(*g*_1_,*q*_1_,*r*_1_) ≥ *U*_*TS*_ and under universal suffrage it is *v*_*L*_(*g*_1_) ≥ *U*_*US*_.

Proposition 1 The capitalist-politician generates a rational political budget cycle.

1. Taxpayer suffrage: the pre-election budget is *r*_*TS*_ < r*, *g*_*TS*_ = g*, *q*_*TS*_ < q* and *τ*_*TS*_ < τ*.
2. Universal suffrage: the pre-election budget is *r*_*US*_ = r*, *g*_*US*_ > g*, *q*_*TS*_ < q* and $\tau_{\mathrm{TS}}\begin{array}{c} \geq \\ < \end{array}\tau^{*}$.

The capitalist-politician wants more rents and more spending on the non-productive public good than do capitalist-voters. Under taxpayer suffrage, the capitalist-politician cuts spending on the non-productive public good and rents to convince capitalist-voters of his quality. Since all capitalists agree on the optimal level of the productive public good, there is no pre-election distortion in that item. The combined consequence is that the tax rate falls. In short, the rational political budget cycle manifests itself as pre-election cuts in rents, less spending on non-productive public goods and lower taxes. We call this the retrenchment hypothesis.

Under universal suffrage, worker-voters want more spending on the productive public good than the capitalist-politician. They are not concerned with the other budget items because the incidence of the property tax is passed on and because they do not benefit from non-productive public goods. Consequently, the capitalist-politician delivers the utility target *U_US_* by spending more on the productive public good. The maximum rent is then extracted and spending on the non-productive public good is cut. The reason for the latter is that the increase in spending on *g* decreases net profit income, making it optimal to reduce spending on *q*. The net effect on the tax rate is ambiguous. In short, under universal suffrage the rational political budget cycle manifests itself as a pre-election hike in spending on productive public goods and a cut in non-productive services, with an uncertain effect on taxes. We call this the expenditure switching hypothesis.

The LMBs could not run deficits but surpluses could be accumulated for precautionary reasons.^18^ We can capture this by assuming that the incumbent politician has a surplus target in non-election years but may deviate from this in election years (at a cost). Under taxpayer suffrage, the benefit is that more rents can be retained. Under universal suffrage, some of the increase in spending on productive public goods can be financed by suspending the surplus target. Surpluses may, therefore, be lower in election than in non-election years irrespective of the suffrage rules. This is the third hypothesis we test.

## 4 Data

Table 1 lists the 28 London Metropolitan Boroughs and the ID number used to identify each of them in the maps shown below. Information on the LMBs' accounts is published in the Local Taxation Returns (1901–1914) and in the Local Government Financial Statistics (1920–1938). These sources contain detailed information on income, expenditures (current and capital) and debt for each borough. The format of the accounts, however, changed significantly after the First World War, when the responsibility for collecting and reporting local government public finance data moved from the Local Government Board to the Ministry of Health. After this change, a greater emphasis was put on recording information related to public health. This makes it impossible to match disaggregated budget items between the two sources and we consider two separate samples, corresponding to the two suffrage regimes. We stress, however, that a careful reading of the notes to the accounts gives us no reason to believe that there were any substantial alterations to LMB accounting practises that could account for systematic differences in the nature of the political budget cycle before and after the change in suffrage rules.

The fiscal year runs from April 1 to March 31 throughout and we use the convention to refer to a fiscal year by the calendar year in which it ends. The taxpayer suffrage sample runs from 1902 to 1914. We cannot use the data for 1901 because the accounts only refer to a part of the year (November 1900 to March 1901) and 1914 is the last fiscal year available since systematic reporting was suspended during much of the War. The first accounts after the War for the fiscal year 1920 were incomplete and are excluded from the analysis. The universal suffrage sample, therefore, starts with the fiscal year ending in 1921 and runs to 1937. This gives a total of 364 observations for the taxpayer suffrage sample and 476 for the universal suffrage sample.^19^ The fiscal data is converted into real values using the Sauerbeck-Statisk price index from Mitchell (1988) with base year 1871 and expressed in per 1000 capita terms. The seven particular fiscal outcomes that we study are listed and defined in Table 2.

Elections took place every three years: 1900, 1903, 1906, 1909 and 1912 before the War; and 1919, 1922, 1925, 1928, 1931, 1934, and 1937 after. The potential manipulation of the budget would occur before the election and would therefore fall in the fiscal year spanning the November election. We define the dummy variable *election* as being equal to one if fiscal year *t* is an election year and zero otherwise.

**Fig. 1:**
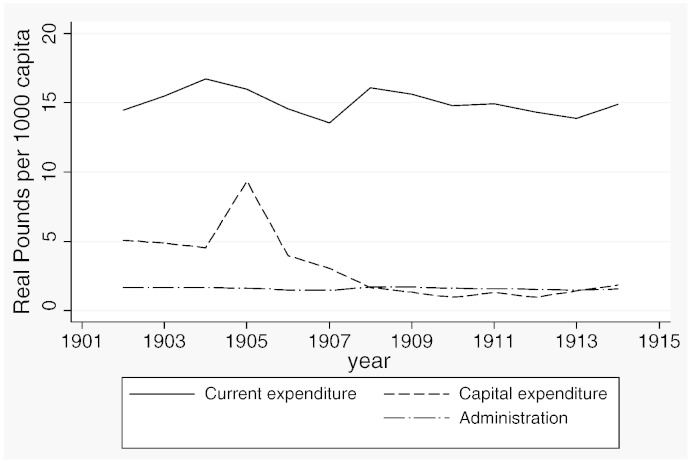
Expenditure outcomes, 1902–1914.

**Fig. 2:**
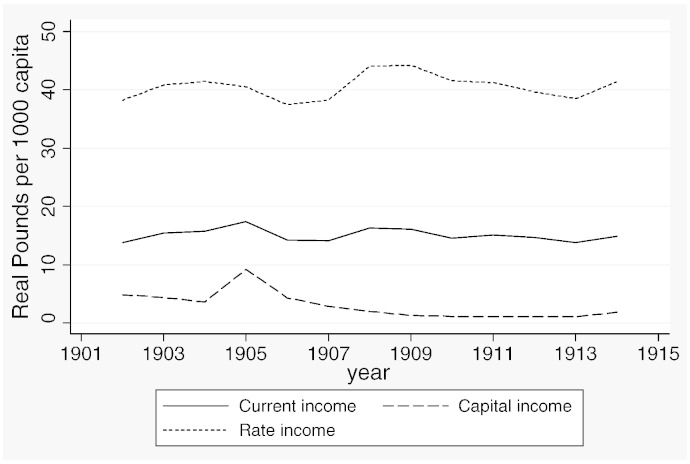
Revenue outcomes, 1902–1914.

**Fig. 3:**
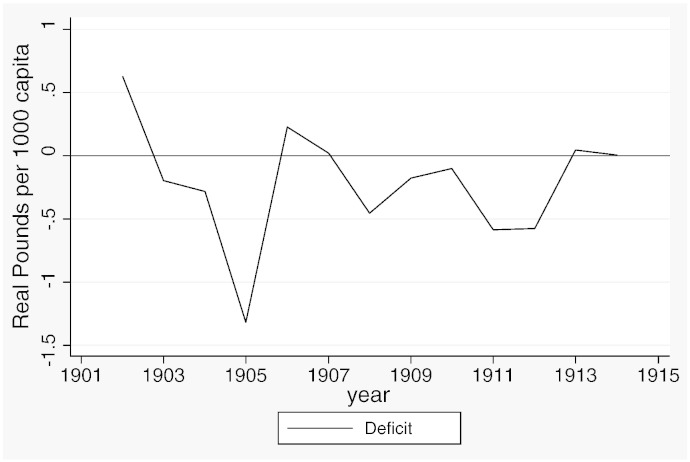
Current deficit, 1902–1914.

**Fig. 4:**
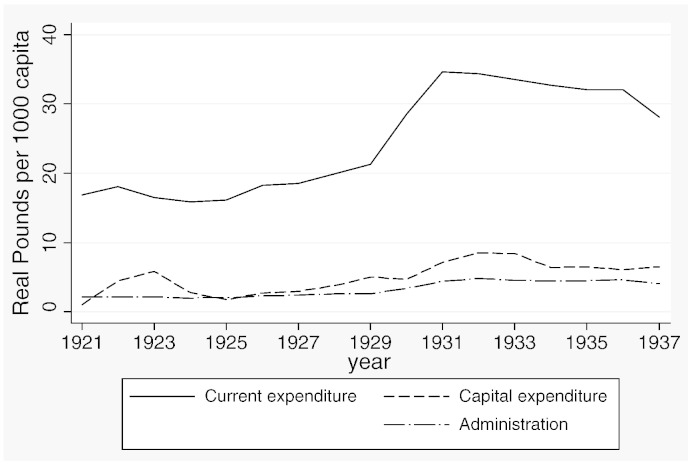
Expenditure outcomes, 1921–1937.

**Fig. 5:**
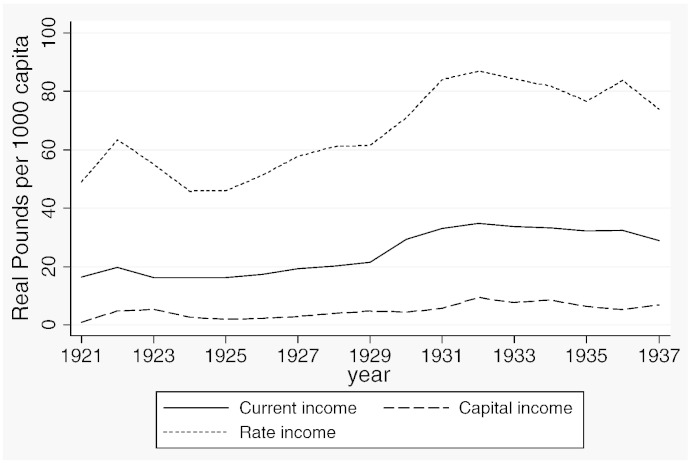
Revenue outcomes, 1921–1937.

**Fig. 6:**
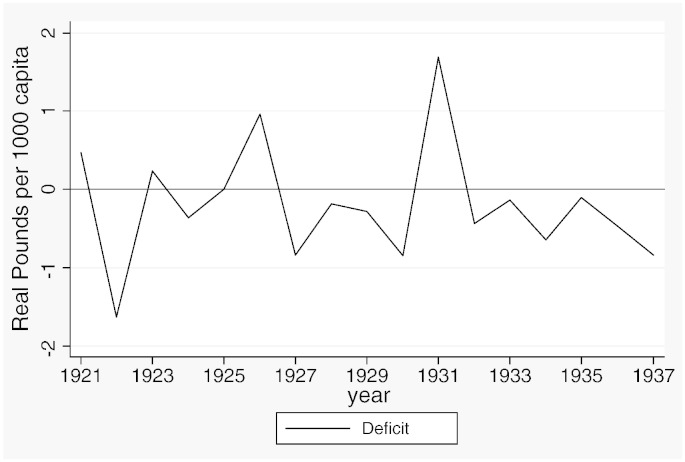
Current deficit, 1921–1937.

Table 3 reports descriptive statistics separately for the two samples and Figs. 1–6 show the average trends for the fiscal outcome variables for the fiscal years 1902–14 and 1921–37, respectively. We notice a number of important facts. First, both *current income* and *current expenditure* increase in real terms from around £15 per 1000 capita (in 1871 prices) under taxpayer suffrage to £25 per 1000 capita under universal suffrage (see Table 3). The increase in *capital expenditure* (and *capital income*) is less pronounced. Secondly, there were no particular trends in *current expenditure* or in spending on *administration* under taxpayer suffrage (Fig. 1). Likewise, *current income* and *rate income* are stable in this period (Fig. 2). A similar characterization applies to the trends under universal suffrage (Figs. 4 and 5) and we note that, on average, the LMBs' spending and taxation levels were comparable in 1914 and 1921, despite the interruption of the War and the franchise change. We do, however, observe a decline in *capital expenditure* (and *capital income*) under taxpayer suffrage in the years before the War (Figs. 1 and 2). The spike in *capital expenditure* in 1905 is entirely attributed to a large investment in electricity in St. Marylebone and is (more than) matched by a large increase in *capital income* (a big loan). Thirdly, around 1930, a marked level shift upwards in *current expenditure* and in *rate income* but not in *capital expenditure*, takes place. A disaggregated analysis of the data [not reported] suggests that this reflects increases in spending on streets as well as increases in wage costs. Gillespie (1989) documents how some boroughs in the 1920s used resources for public relief work and we conjecture that this endeavour was intensified during the recession years. Fourthly, we observe substantial year-on-year variation in the average *current deficit* (Figs. 3 and 6). Mostly the LMBs were close to balancing the books and, on average, they ran a small surplus both before and after the change in the franchise (see Table 3). This suggests that the balanced budget rule mattered, but, at the same time, allowed some flexibility for fiscal manipulations.

**Map 1:**
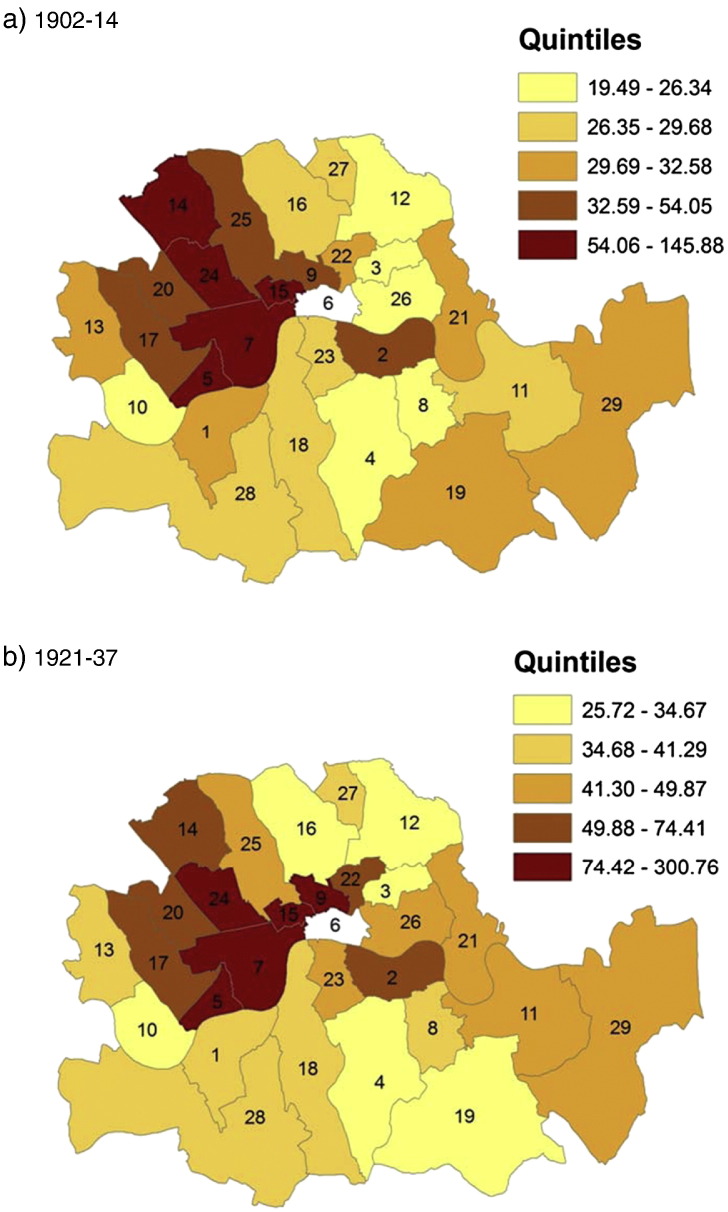
Rate income (£ per 1000 capita) in London Metropolitan Boroughs before and after World War I.

**Map 2:**
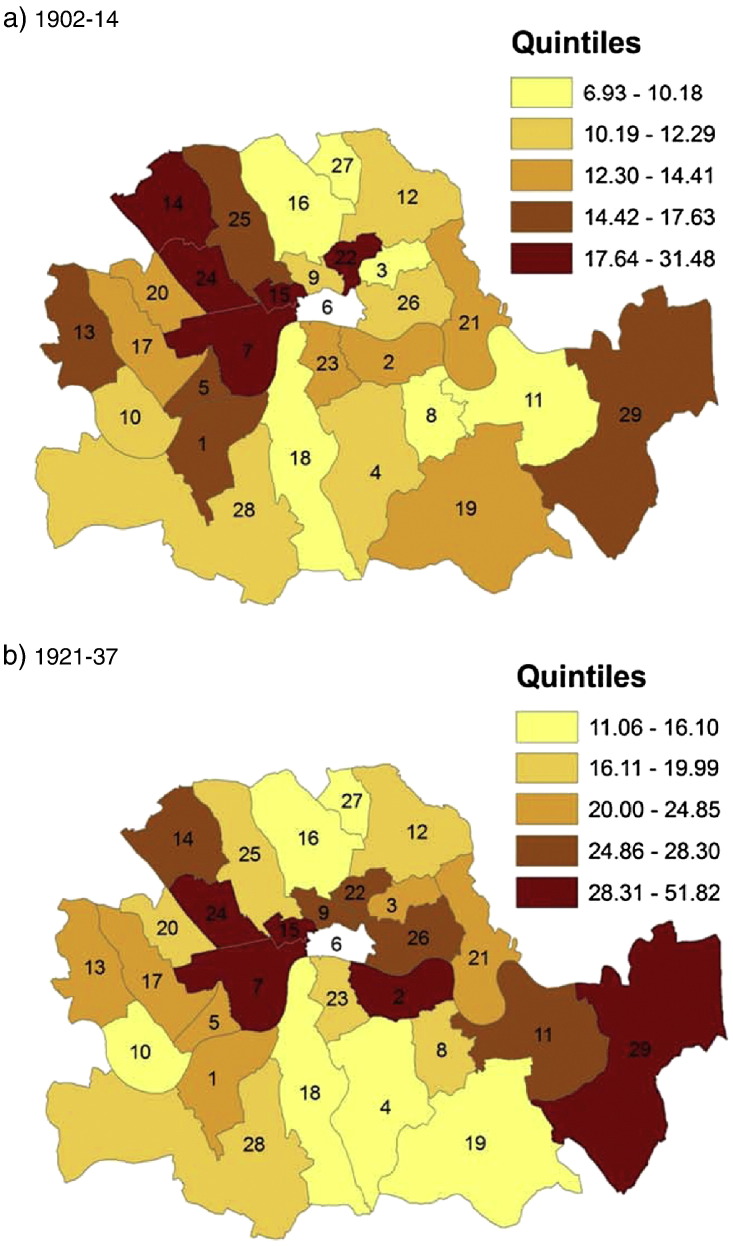
Current expenditure (£ per 1000 capita) in London Metropolitan Boroughs before and after World War I.

**Map 3:**
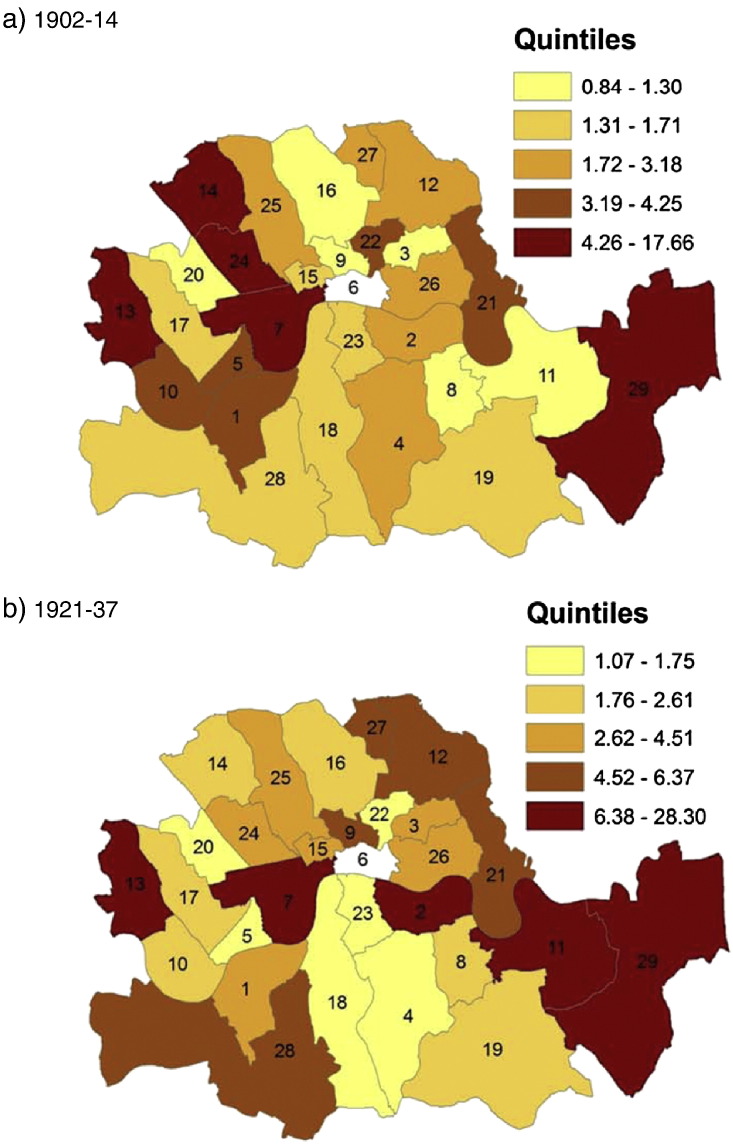
Capital expenditure (£ per 1000 capita) in London Metropolitan Boroughs before and after World War I.

The average trends hide substantial cross sectional variation: some boroughs spent, taxed and borrowed much more than others. The dispersion is particularly large with regard to capital expenditures (and income) where the standard deviation is about twice as large as the mean values (see Table 3). We visualize this dispersion in Maps 1–3. Each map consists of two panels, one for the pre-war and for the post-war period, and colour codes the spatial distribution of *rate income* (Map 1), *current expenditure* (Map 2) and *capital expenditure* (Map 3). There is a consistent spatial pattern of high-tax–high-current-spending in north-west London, including Westminster, Holborn, St. Marylebone, and Hampstead, and Woolwich in the south-east. These are also the areas with high levels of capital expenditure under taxpayer suffrage. After the change to universal suffrage in 1918 there is a marked shift in *capital expenditure* to the east and south-east of London, with Poplar, Bermondsey and Greenwich standing out as big spenders. Much political debate was generated after the First World War about the high-rating and -spending policies of east end Labour councils such as Poplar. Leaders of the Labour Party in London were worried that this approach would alienate potential middle-class support in other parts of the capital (Gillespie, 1989).

We also collect demographic data – total population, population growth (absolute change in number of inhabitants), population density (inhabitants per house) and age structure (proportion of the population below 20) – from the decennial Censuses.^20^ We do not have income or GDP data for the boroughs, but we record the average value of properties subject to taxation in each borough each year and use the variable *wealth* (defined as taxable value per 1000 houses) to proxy for income or wealth effects.^21^ We record information on the stock of outstanding loans at the end of each fiscal year and use the variable *debt* (defined as outstanding real debt per capita), as a proxy for accumulated spending on public services. Finally, for the taxpayer suffrage sample, we have collected information on the number of registered voters in each borough. We normalize this with the size of the adult male population to get the variable *franchise extension* which we use to control for variations in the size of the electorate. We have collected a number of additional variables used for robustness checks. We introduce these in Section 8.

## 5 Evidence for the political budget cycle

The fiscal outcome variables defined in Table 2 are selected to facilitate tests of the retrenchment and expenditure switching hypotheses. Retrenchment effects would primarily show up as election year tax cuts. This is captured by *rate income* and *current income* where the latter, in addition to property tax revenue, includes income from user charges for local public services, but excludes revenues raised on behalf of other local authorities. Expenditure switching involves increasing spending on productive public goods that benefits all and cuts in non-productive spending. We presume that the outputs generated by capital expenditures represent productive public spending. In contrast, many current spending items are non-productive. Therefore, we use the variables *capital expenditure* and *current expenditure* to test the expenditure switching hypothesis. In addition, we use the variable *capital income* to test if there is a tendency to take out loans in election years. If this is the case, the need to increase the yield from property taxes to fund the pre-election spending hike in capital spending anticipated under universal suffrage would be reduced and we might expect to see a fall in tax income to match the expected fall in current spending. We use expenditure on *administration* as a proxy for bureaucratic spending with the rationale that the taxpayer-voter might find such outlays particularly wasteful.^22^ Finally, we use *current deficit*, defined as total current expenditure minus current income, to test election cycles in the fiscal balance.

**Fig. 7:**
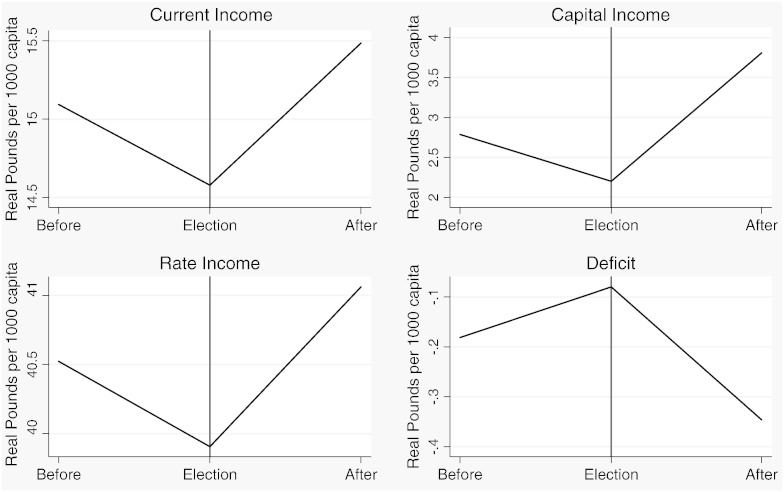
The PBC in revenue outcomes, 1902–1914.

**Fig. 8:**
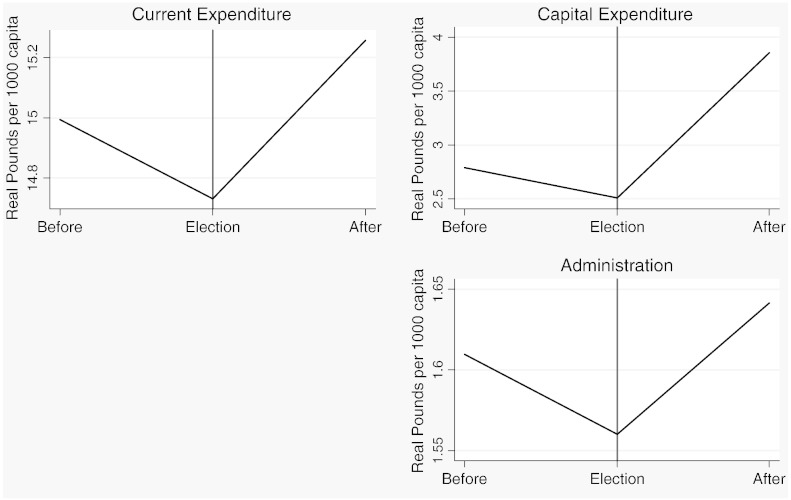
The PBC in expenditure outcomes, 1902–1914.

**Fig. 9:**
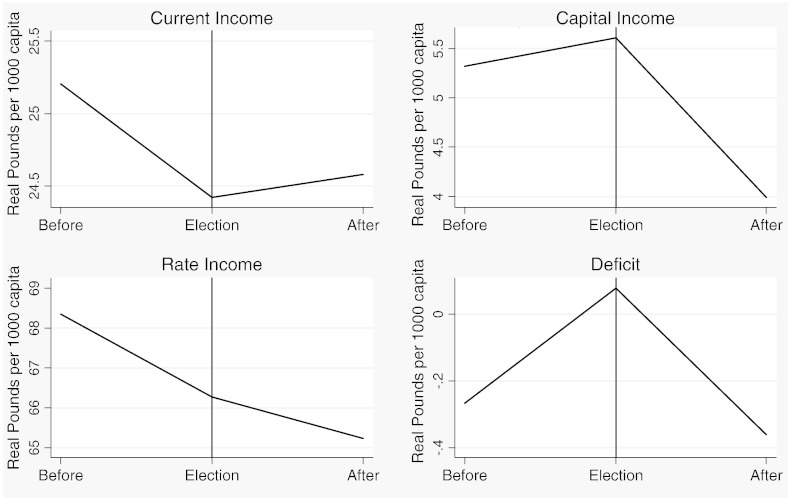
The PBC in revenue outcomes, 1921–1937.

**Fig. 10:**
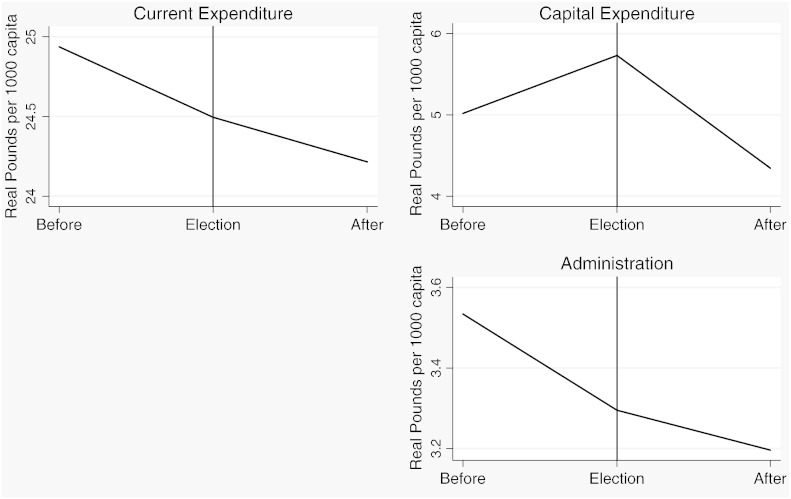
The PBC in expenditure outcomes, 1921–1937.

Before we turn to the formal statistical analysis, we present some descriptive evidence on the nature of the opportunistic political budget cycle in London between 1902 and 1937. Figs. 7–10 show plots of the seven fiscal outcome variables in “event time”. That is, each figure shows the average of the relevant fiscal outcome in election years, one year before an election and one year after an election. A “V” or an inverted “V” shape indicates a political budget cycle. We observe a clear revenue pattern under taxpayer suffrage: lower *current income* and lower *rate income* in election years than in other years (Fig. 7). We note a fall in spending for both *administration* and *current expenditure* (Fig. 8). The pattern is noticeably different under universal suffrage (Figs. 9 and 10). The budget cycle in *rate income* has gone. Instead, we observe a clear election year increase in *capital expenditure* with a hint of a cycle in *current income*. Under both franchise regimes, surpluses are lower in election years.

Altogether, it appears that the political budget cycle differed before and after the expansion of the franchise in ways that are consistent with the retrenchment and expenditure switching hypotheses. It is clear, of course, that many other factors than the differences in the suffrage rules could be behind this, including the political ideology of the councils' governing parties and macro-economic trends such as the Great Depression. We consider these and other potential influences in a later section. First, however, we turn to a systematic analysis of the data.

## 6 Empirical specification

As in Brender and Drazen (2005), Shi and Svensson (2006), Veiga and Veiga (2007) and many other studies of the political budget cycle, we estimate a partial adjustment model of the following type:

(7) $$Y_{\mathrm{it}}=\alpha_{i}+\beta_{1}Y_{\mathrm{it}-1}+\beta_{2}\mathrm{electio}n_{t}+X_{\mathrm{it}}\gamma +\epsilon_{\mathrm{it}},$$

where *Y_it_* is a particular fiscal outcome in year *t* in borough *i*, *election_t_* is the election year dummy variable, and *ϵ_it_* is an error term. The vector *X_it_* contains the demographic control variables (*population*, *population growth*, *population density* and *age structure*) and the proxy for income, *wealth*. In addition, for specifications where the outcome variable is a spending item, we control for the stock of outstanding loans in order to proxy for past investments (*debt*). For the purpose of analysing the taxpayer suffrage sample, we include the measure of the fraction of adult males who were registered as voters (*franchise extension*). We include borough fixed effects to capture time invariant characteristics of the boroughs.^23^ The timing of the elections is exogenous so we need not worry about the endogeneity of elections or that the timing might be chosen strategically to win elections. As already noted, we study the two suffrage regimes separately as two different samples and thus allow *β*_2_ (along with all the other parameters of the model) to vary with the suffrage regime.

In an attempt to balance various econometric issues with the data at hand, our model uses two different estimators. The first is a fixed effect estimator. We cluster the standard errors at the borough level to take into account the fact that autocorrelation in a fixed effect model may inflate the z-statistics and cause invalid inference (Bertrand et al., 2004). The lagged dependent variable may, however, cause a Nickell bias (Nickell, 1981), since our two samples have only 12 and 16 years of observations, respectively.^24^ Our second estimator takes this into account. The GMM estimator (Blundell and Bond, 1998) used by, e.g., Shi and Svensson (2006), is not ideal in our case as it requires many more cross sectional units to yield consistent estimates than we obtained. For this reason, we use the bias-corrected least-squares dummy variable (LSDV) estimator. It performs better than the GMM estimator in panels with a small cross section (Bruno, 2005a,b).

## 7 Results

The main results for the taxpayer suffrage sample are recorded in Table 4 (revenue outcomes) and Table 5 (expenditure outcomes). The corresponding results for the universal suffrage sample are reported in Tables 6 and 7. For each fiscal outcome, we report both the estimates obtained with the fixed effects and the LSDV estimator, which mostly yield similar results for the election year indicator variable. We find strong evidence of an opportunistic political budget cycle in both samples but the nature of the cycle is conditional on the suffrage rules.

Under taxpayer suffrage, the political budget cycle shows up as a reduction in *current income* and in *rate income* in the election year (Table 4). Spending on *administration* is cut in election years. There is no detectable impact on the relative composition of capital and current spending (Table 5). The cut in *administration* is insufficient to balance the books and we find that election years are associated with lower surpluses (Table 4). In other words, the election year tax cut is partly funded by cutting back on bureaucracy and partly by running a smaller surplus (or a small “unplanned” deficit). The magnitude of the tax cut is about £0.6 per 1000 capita which should be compared to the total income (net of precept) of £15 per 1000 capita. The reduction in *administration* corresponds to about a 1.5 per cent cut in the election year. These are sizable effects which are consistent with the retrenchment hypothesis.

We observe a different pattern under universal suffrage. Most notable are election year increases in *capital expenditure* and reductions in *current expenditures* (Table 7). The increase in *capital expenditure* is £0.93 per 1000 capita with the average *capital expenditure* being about £5.2 per 1000 capita. The reduction in *current expenditure* is somewhat smaller (£0.55 per 1000 capita with average expenditure being £25). This suggests that the LMBs systematically moved large-scale capital projects to the election year, while cutting back on current spending. On the revenue side, we find no evidence of a political budget cycle in *tax income* or in *capital income*. There was an election year drop, however, in *current income* (Table 6) and a tendency to run smaller surpluses or larger deficits in election years. Since *rate income* is unaffected, the fall in *current income* can be attributed to election year reductions in user chargers. These findings are consistent with the expenditure switching hypothesis.

The estimations yield some additional results which are of independent interest. Firstly, the variable *wealth* is positively related to current spending and revenues in both samples. This is consistent with Wagner's Law that relates the size of government to income and wealth (Wagner, 1883). Secondly, insofar as the variable *population* captures scale effects, we notice that the negative point estimate on this variable in the estimations with current (and sometimes also with capital) expenditure is consistent with decreasing returns to scale in the production of these services. Millward and Sheard (1995), in their study of the local finances of 25 provincial municipalities in England and Wales from 1870 to 1914, also find evidence of (moderate) diminishing returns. Population growth correlates negatively with revenue and expenditure outcomes, but is only significant in the universal suffrage sample. In the taxpayer suffrage sample, we control for the size of the electorate with the variable *franchise extension*. The boroughs which experienced an extension of the suffrage (due, for example, to changes in the fraction of property rated for tax purposes) tended to collect more tax income and to run smaller surpluses (Table 4).

## 8 Robustness checks

In this section, we discuss a number of robustness checks and evaluate some alternative interpretations of our findings.

### 8.1 Partisan cycles

The opportunistic political budget cycles that we have emphasised above do not make a distinction between the ideologies of the political parties in power but simply assume that all parties are primarily interested in getting re-elected. There exists, however, a well-established literature, beginning with the classical work by Hibbs (1977), Chappell and Keech (1986), and Alesina (1987), which takes the view that partisan cycles in economic and fiscal outcomes can emerge because parties have different views on appropriate policies and their hold on power fluctuates. In the context of budget cycles, the relevant distinction is between parties on the left which support higher spending and, with a balanced budget rule, higher taxes; and parties on the right which support lower spending and taxation. Both before and after the First World War, elections in London were fought along partisan lines (White, 2001). Before the War, the Progressive Party and the Moderate Party were the two dominant parties in local elections in London. At the time, the Progressive Party consisted of a mixture of Liberals, Fabian socialists and radicals and it generally favoured high (local) government spending. The Moderate Party and, from 1906, The Municipal Reform Party, created by Conservatives and Unionists, were the dominant right-wing parties. The political landscape changed after the First World War with the increased prominence of the Labour Party. This undoubtedly had its source in the Representation of the People Act of 1918 which enfranchised the working-class and gave it a strong voter base. Consequently, the Labour Party replaced the Progressive Party as the dominant left-wing party. The right-wing opposition formed a secret anti-Labour pact in 1922 in response to the popularity of the Labour Party in the first election after the War.

To investigate whether partisan cycles were important, we code the dummy variable *left* for years in which a notionally left-wing party (the Moderate Party, the Labour Party or, in one case, the Socialist Party) holds the majority in the borough council and zero otherwise.^25^ We conjecture that, if anything, spending and taxation levels should be higher during the term of a left-wing party. The results are reported in Panel A of Tables 8 and 9 for the taxpayer suffrage and universal suffrage sample, respectively. The variable *left* is not significant except for one of the fiscal outcomes: for the taxpayer suffrage sample, *left* has a positive effect on current expenditures (at the 10 per cent level of significance). The evidence for partisan cycles is clearly weak, justifying our focus on opportunistic cycles. Importantly, controlling for ideology does not affect the evidence for opportunistic cycles at all: the rise of the Labour Party after the First World War cannot by itself explain the observed difference in the opportunistic political budget cycle.

### 8.2 Absentee owners

Under taxpayer suffrage, owners of rated property in a borough were eligible to vote even if they did not reside in that borough. These absentee voters did not enjoy the benefits of better local public services to the same extent as resident voters. Accordingly, they might have been particularly inclined to support retrenchment and economy. It is, therefore, possible that variations in the fraction of absentee owners could by itself affect fiscal outcomes under taxpayer suffrage. Unfortunately, we do not have information on the number of absentee owners/voters, so we use data from the Censuses of 1901, 1911 and 1921 on the number of uninhabited houses as a proxy. The number of uninhabited houses in the boroughs ranged from 199 to 3283. We have no way of testing how strong the correlation between empty property and absentee owners is. Nonetheless we see from Panel B of Table 8 that the variable *absentee owners* is insignificant for all fiscal outcomes and that evidence of the opportunistic political budget cycle is as before.

### 8.3 The Great Depression

The economic climate in the 1930s was very different to that in the first decade of the century and in the 1920s. It is possible, therefore, that the Great Depression, and not the change from taxpayer to universal suffrage, could explain the differences in the nature of the budget cycle that we observe between the two sample periods. It should be mentioned here that London and the South East fared comparatively well during the Great Depression (White, 2001). There were pockets of extremely high unemployment in the capital but the overall unemployment rate in London was low (Marriott, 1991). As Booth and Glynn (1975) observed, when national insured unemployment peaked in 1932 at 22.1%, it was 13.5% in London, 28.5% in the North East, and 36.5% in Wales. Similarly, Hatton (2003) noted that across the period 1923–1938, average regional rates of unemployment varied from 8% in London and the South East to around 22% in parts of Wales and Northern Ireland. It is therefore unsurprising that White has written that the “1920s and 1930s consolidated the rise in the standard of life of the London working class that the First World War had so unexpectedly fan-fared” (White, 2001, p. 226).

We recall from Figs. 4 and 5 that *current spending* and *tax income* shift upwards around 1930, suggesting that the depression years triggered a fiscal expansion amongst the LMBs. To investigate if the Great Depression and the associated jump in spending and taxation contributed to shaping the political budget cycle during the interwar years, we have re-estimated the model for the universal suffrage sample with the inclusion of a dummy variable, *Great Depression*, coded one for depression years from 1929 to 1937 and zero otherwise. The results are reported in Panel B of Table 9. The dummy variable is positive and significant, as expected, for *current spending*, *current income* and *tax income*. The depression years were, on average, associated with larger surpluses. More importantly, the evidence for the political budget cycle from Tables 6 and 7 is robust after controlling for *Great Depression*. We conclude from this that the Great Depression did exert some influence on the public finances of the LMBs but was not itself responsible for the difference in the nature of the political budget cycle before and after the extension of the franchise.

### 8.4 Heterogeneous election year effects

The baseline results concern the average election year effect across the 28 LMBs in the two samples. This may mask important heterogeneity. To investigate this, we have re-estimated the baseline specification with a set of 28 borough-specific election year dummy variables. The results are summarized in Tables 10 and 11 which report the coefficient on the election year dummy for each borough for the two samples. We observe some heterogeneity as one would expect, but there is no indication that the average results are driven by one or two outliers. Table 10, with the results from the taxpayer suffrage, is sorted according to the size of the electorate (*franchise extension*). While the point estimates on the vast majority of borough-specific election year effects in the *current income* and *tax income* regressions are negative and significant, there are a few boroughs where the effect is positive. These are concentrated in boroughs with the most restricted franchise. This is consistent with the finding in Aidt et al. (2010) that retrenchment is most pronounced where penny-conscious middle class voters gain control of the councils.

### 8.5 Other robustness checks

We have checked whether the null result for *capital expenditure* and *capital income* in the taxpayer suffrage sample can be attributed to the large investment recorded for St. Marylebone in 1905. Excluding this borough from the sample does not affect any of the results [not reported].

## 9 Conclusion

The evidence base for the existence of political budget cycles is overwhelming. Politicians use the fiscal levers granted to them to win re-election if they can. How this plays out is, unsurprisingly, a function of the institutional constraints imposed on the elected representatives. As pointed out by Brender and Drazen (2005), Shi and Svensson (2006) and many others, the political budget cycle is *conditional*. We contribute to the literature on the conditional political budget cycle in two main ways. Firstly, the focus of previous research has been on the period after the Second World War. In contrast, we enlist data from the early part of the 20th century and find that the political budget cycle is by no means a recent phenomenon: it was alive and kicking in London both in the years leading up to the First World War and during the interwar period. Secondly, precisely because of the emphasis on modern data, previous research explored the political budget cycle in the context of universal suffrage.^26^ Our historical perspective allows us to investigate the nature of the cycle under two different suffrage regimes and we find that it differs in marked but predictable ways.

This strengthens the existing evidence base that the political budget cycle is contingent on political institutions and rules. Persson and Tabellini (2003), Streb et al. (2009), and Klomp and de Haan (2012) have previously demonstrated that election rules, regime types and legislative checks and balances affect the nature of political budget cycles in modern democracies. Gonzalez (2002) finds that the political budget cycle in Mexico was magnified as democratic institutions improve in quality while Potrafke (2012) reports that the cycle is stronger under two- rather than under multi-party systems. Others have found that the experience of voters, information flows and fiscal transparency are also important.^27^ The picture that emerges from this literature has yet to come into sharp focus, but one lesson is clear: context is crucial for the incentive and ability of incumbent politicians to manipulate expenditure and taxes for electoral gain. We have added new evidence to the understanding of the conditional nature of the political budget cycle by demonstrating that the suffrage rules themselves matter.

## Figures and Tables

**Table 1:** The London Metropolitan Boroughs.

Name	ID number	Name	ID number
Battersea	1	Islington	16
Bermondsey	2	Kensington	17
Bethnal Green	3	Lambeth	18
Camberwell	4	Lewisham	19
Chelsea	5	Paddington	20
City of Westminster	7	Poplar	21
Deptford	8	Shoreditch	22
Finsbury	9	Southwark	23
Fulham	10	St. Marylebone	24
Greenwich	11	St. Pancras	25
Hackney	12	Stepney	26
Hammersmith	13	Stoke Newington	27
Hampstead	14	Wandsworth	28
Holborn	15	Woolwich	29

Note: The City of London, which was not a Metropolitan Borough, is not included in the analysis. It has the ID number 6 and is shaded white in all the maps.

**Table 2:** Definitions of the eight fiscal outcome variables.

Variable name	Definition Taxpayer suffrage	Definition Universal suffrage
Current income	Receipts from the rates, user charges and grants other than from loans, net of payments under precept^a^ to other local authorities.	Annual income of the rate fund and general services, net of payments under precept.
Capital income	Receipts from loans.	Capital receipts (including loans) for the rate fund and general services maintained by the borough council.
Rate income^b^	Receipts from general and other rates including funds raised to meet precept.	Income from public rates (general and other) including funds raised to meet precepts.
Current expenditure	Spending on services such as streets, refuse collection, public lighting, sewers and drainage, public works, burial grounds, baths and washhouses, loan charges and salaries & administration, excluding payments under precept to other local authorities.	Annual expenditure on general services (the same type of spending as under taxpayer suffrage).
Capital expenditure	Investments on depots and refuse, made under the electricity act, on streets, on housing, on parks, on public buildings, on sewerage and drainage, on baths and washhouses, and on public libraries.	Capital expenditures on general services (the same type of investments as under taxpayer suffrage).
Administration^b^	Current expenditure on spending on salaries and other remuneration of officers and establishment charges.^c^	Spending on medical officers, total administrative expenses and rate collection expenses.
Deficit	Current expenditure minus current income.	Current expenditure minus current income.

Note: Source: The Local Taxation Returns (1901–1914) and the Local Government Financial Statistics (1918–1938).

a Precept is the tax payment collected by the council for other local authorities (the Boards of Guardians, London County Council, the School Board for London and the Metropolitan Police).

b For the universal suffrage sample the data are available from 1923.

c Many administrative expenses were allocated directly to the services that they helped provide and cannot be separated out.

**Table 3:** Descriptive statistics.

Variable name	Average	Std. Dev.	Minimum	Maximum	Average	Std. Dev.	Minimum	Maximum
	1902–1914				1921–1937			
Current income	15.08	7.01	4.42	49.49	25.28	13.34	4.99	79.83
Capital income	3.00	8.69	0	155.58	5.17	8.13	0	55.31
Rate income	40.54	25.18	17.12	164.61	67.74	63.62	11.71	434.22
Current expenditure	15.01	6.84	5.73	49.83	25.04	13.13	4.93	79.46
Capital expenditure	3.11	8.39	0	149.87	5.23	7.85	0	50.11
Administration	1.60	0.64	0.69	4.17	3.41	2.04	0.59	11.59
Deficit	− 0.21	1.41	− 6.98	8.78	− 0.24	2.05	− 12.77	13.32
Population	160,829	75,342	47,508	334,232	160,025	97,528	34,850	901,000
Population growth	− 47	1724	− 3015	7936	601	11,264	− 3666	96,534
Population density	14.15	2.07	8.56	19.83	16.64	2.21	4.84	21.32
Age structure	37.87	5.47	24.89	46.53	32.41	5.61	20.47	43.68
Wealth	843.60	618.44	329.29	4053.25	735.42	774.63	214.06	5566.17
Debt	34.73	34.36	5.28	235.93	66.77	67.99	2.87	374.79
Franchise extension	61.7	8.8	36.3	81.8	n.a.	n.a.	n.a.	n.a.
Left	0.23	0.42	0	1	0.29	0.45	0	1
Absent owners	1190	610	199	3283	n.a.	n.a.	n.a.	n.a.

Note: See Table 2 and the text for definitions of the variables. All fiscal variables are in real 1871 Pounds per 1000 capita.

**Table 4:** Estimation results for revenue outcomes and current deficit for the taxpayer suffrage sample, 1902–1914.

Variables	(1)	(2)	(3)	(4)	(5)	(6)	(7)	(8)
	Current income	Current income	Capital income	Capital income	Rate income	Rate income	Deficit	Deficit
Lagged dep. var.	0.41⁎⁎⁎	0.48⁎⁎⁎	− 0.0022	0.099	0.33⁎⁎⁎	0.38⁎⁎⁎	− 0.10⁎⁎	− 0.036
	[3.18]	[9.89]	[− 0.054]	[1.52]	[5.32]	[7.81]	[− 2.74]	[− 0.56]
Election	− 0.47⁎⁎⁎	− 0.51⁎⁎⁎	− 1.57	− 1.62⁎⁎	− 0.50⁎⁎	− 0.51⁎⁎	0.36⁎⁎	0.38⁎⁎⁎
	[− 3.24]	[− 2.74]	[− 1.30]	[− 2.06]	[− 2.56]	[− 2.23]	[2.76]	[2.96]
Franchise extension	0.040	0.041	− 0.12	− 0.11	0.074⁎⁎⁎	0.080⁎⁎	0.046⁎⁎⁎	0.044⁎⁎
	[1.53]	[1.46]	[− 1.53]	[− 0.95]	[2.79]	[2.22]	[2.77]	[2.39]
Population growth	− 0.000060	− 0.000078	0.000041	0.000049	− 0.000021	− 0.000021	0.000056	0.000067
	[− 0.54]	[− 0.32]	[0.15]	[0.048]	[− 0.075]	[− 0.067]	[0.91]	[0.42]
Population	− 0.000085⁎⁎⁎	− 0.000079⁎⁎⁎	0.000082	0.000082	− 0.00010⁎	− 0.00010⁎⁎⁎	4.3e − 06	5.0e − 06
	[− 4.70]	[− 3.13]	[0.96]	[0.77]	[− 2.01]	[− 3.15]	[0.32]	[0.30]
Age structure	− 0.099	− 0.10	1.24⁎⁎	1.13	0.35	0.32	0.088	0.081
	[− 0.48]	[− 0.42]	[2.24]	[1.08]	[0.93]	[1.05]	[0.60]	[0.51]
Population density	0.69⁎⁎	0.67⁎⁎⁎	− 2.40	− 2.38⁎⁎	3.06⁎⁎⁎	2.91⁎⁎⁎	0.045	0.064
	[2.16]	[2.60]	[− 1.31]	[− 2.16]	[3.70]	[8.49]	[0.33]	[0.37]
Wealth	0.015⁎⁎⁎	0.014⁎⁎⁎	− 0.017	− 0.017⁎⁎	0.025⁎⁎⁎	0.025⁎⁎⁎	− 0.00016	− 0.000093
	[6.92]	[8.37]	[− 1.16]	[− 2.35]	[10.5]	[11.4]	[− 0.075]	[− 0.080]
Estimation method	Fixed effects^a^	LSDV^b^	Fixed effects^a^	LSDV^b^	Fixed effects^a^	LSDV^b^	Fixed effects^a^	LSDV^b^
Observations	336	336	336	336	336	336	336	336
R-squared	0.45		0.06		0.53		0.08	
Number of boroughs	28	28	28	28	28	28	28	28

Note: All fiscal variables are expressed in real Pounds per 1000 capita.

a Robust z-statistics clustered at the borough level in brackets; borough fixed effects included.

b Bias corrected LSDV dynamic panel data estimator suggested by Bruno (2005a,b).

⁎⁎⁎ p < 0.01.

⁎⁎ p < 0.05.

⁎ p < 0.1.

**Table 5:** Estimation results for expenditure outcomes for the taxpayer suffrage sample, 1902–1914.

VARIABLES	(1)	(2)	(3)	(4)	(5)	(6)
	Current expenditure	Current expenditure	Capital expenditure	Capital expenditure	Administration	Administration
Lagged dep. var.	0.23⁎⁎⁎	0.16⁎⁎⁎	0.063	0.16⁎⁎	0.44⁎⁎⁎	0.49⁎⁎⁎
	[5.34]	[3.10]	[1.01]	[2.29]	[3.99]	[10.0]
Election	− 0.039	− 0.025	− 1.19	− 1.25	− 0.032⁎⁎⁎	− 0.033⁎⁎⁎
	[− 0.18]	[− 0.19]	[− 1.08]	[− 1.54]	[− 4.55]	[− 3.43]
Franchise extension	0.0029	− 0.0065	− 0.12	− 0.11	0.0010	0.0013
	[0.19]	[− 0.33]	[− 1.41]	[− 0.91]	[0.79]	[0.87]
Population growth	− 0.000038	− 0.000062	0.000053	− 0.000017	− 4.1e − 06	− 4.2e − 06
	[− 0.28]	[− 0.37]	[0.16]	[− 0.016]	[− 0.31]	[− 0.34]
Population	− 0.000087⁎⁎⁎	− 0.000089⁎⁎⁎	0.000079	0.000077	− 5.0e − 06⁎⁎⁎	− 4.7e − 06⁎⁎⁎
	[− 6.08]	[− 5.00]	[0.80]	[0.68]	[− 2.89]	[− 3.49]
Age structure	− 0.042	− 0.051	1.18⁎⁎	1.21	− 0.011	− 0.011
	[− 0.20]	[− 0.30]	[2.09]	[1.12]	[− 0.58]	[− 0.88]
Population density	0.48	0.31⁎	− 2.63	− 2.33⁎⁎	0.067⁎⁎	0.067⁎⁎⁎
	[1.26]	[1.69]	[− 1.26]	[− 1.98]	[2.09]	[4.71]
Debt	0.063⁎⁎⁎	0.076⁎⁎⁎	0.027	0.0060	0.00064⁎⁎	0.00054
	[10.4]	[9.56]	[0.99]	[0.14]	[2.26]	[1.06]
Wealth	0.012⁎⁎⁎	0.0090⁎⁎⁎	− 0.018	− 0.017⁎⁎	0.00096⁎⁎⁎	0.00095⁎⁎⁎
	[7.54]	[7.26]	[− 1.11]	[− 2.13]	[14.9]	[10.2]
Estimation method	Fixed effects^a^	LSDV^b^	Fixed effects^a^	LSDV^b^	Fixed effects^a^	LSDV^b^
Observations	336	336	336	336	336	336
R-squared	0.65		0.09		0.60	
Number of boroughs	28	28	28	28	28	28

Note: All fiscal variables are expressed in real Pounds per 1000 capita.

a Robust z-statistics clustered at the borough level in brackets; borough fixed effects included.

b Bias corrected LSDV dynamic panel data estimator suggested by Bruno (2005a,b).

⁎⁎⁎ p < 0.01.

⁎⁎ p < 0.05.

⁎ p < 0.1.

**Table 6:** Estimation results for revenue outcomes and current deficit for the universal suffrage sample, 1921–1937.

Variables	(1)	(2)	(3)	(4)	(5)	(6)	(7)	(8)
	Current income	Current income	Capital income	Capital income	Rate income	Rate income	Deficit	Deficit
Lagged dep. var.	0.75⁎⁎⁎	0.84⁎⁎⁎	0.53⁎⁎⁎	0.61⁎⁎⁎	0.68⁎⁎⁎	0.74⁎⁎⁎	− 0.040	0.017
	[11.4]	[20.1]	[6.54]	[11.6]	[11.2]	[29.9]	[− 0.41]	[0.32]
Election	− 1.01⁎⁎⁎	− 1.09⁎⁎⁎	0.59	0.60	− 0.16	− 0.31	0.47⁎⁎⁎	0.48⁎⁎
	[− 4.47]	[− 2.96]	[1.31]	[1.15]	[− 0.26]	[− 0.31]	[3.14]	[2.46]
Population growth	− 0.000074⁎⁎	− 0.000078⁎	− 0.000040⁎⁎	− 0.000049	− 0.00012⁎⁎	− 0.00015	− 0.00004⁎⁎	− 0.00004
	[− 2.36]	[− 1.68]	[− 2.23]	[− 0.99]	[− 2.57]	[− 1.32]	[− 2.52]	[− 1.48]
Population	− 0.000013	− 0.000014	− 8.2e − 06	− 0.000011	− 8.7e − 07	− 8.2e − 06	0.067	0.063
	[− 1.62]	[− 1.25]	[− 1.12]	[− 0.90]	[− 0.086]	[− 0.29]	[0.33]	[0.22]
Age structure	− 0.98⁎⁎⁎	− 0.72⁎	− 0.40	− 0.42	− 0.42	− 0.42	− 8.7e − 06⁎	− 8.2e − 06
	[− 3.64]	[− 1.94]	[− 1.50]	[− 1.25]	[− 1.04]	[− 0.54]	[− 2.00]	[− 1.41]
Population density	− 1.00	− 1.20⁎	− 0.68	− 0.93	− 0.17	− 1.07	− 0.33⁎⁎	− 0.30⁎
	[− 1.21]	[− 1.91]	[− 1.24]	[− 1.42]	[− 0.18]	[− 0.70]	[− 2.41]	[− 1.72]
Wealth	0.0036⁎	0.0033⁎⁎⁎	0.0036⁎⁎⁎	0.0034⁎⁎⁎	0.021⁎⁎⁎	0.020⁎⁎⁎	− 0.87⁎⁎	− 0.82⁎⁎⁎
	[1.84]	[4.95]	[5.13]	[4.64]	[3.92]	[14.0]	[− 2.26]	[− 2.59]
Estimation method	Fixed effects^a^	LSDV^b^	Fixed effects^a^	LSDV^b^	Fixed effects^a^	LSDV^b^	Fixed effects^a^	LSDV^b^
Observations	448	448	448	448	392^c^	392^c^	448	448
R-squared	0.802		0.379		0.871		0.056	
Number of boroughs	28	28	28	28	28	28	28	28

Note: All fiscal variables are expressed in real Pounds per 1000 capita.

a Robust z-statistics clustered at the borough level in brackets; borough fixed effects included.

b Bias corrected LSDV dynamic panel data estimator suggested by Bruno (2005a,b).

c Sample from 1923 to 1937.

⁎⁎⁎ p < 0.01.

⁎⁎ p < 0.05.

⁎ p < 0.1.

**Table 7:** Estimation results for expenditure outcomes for the universal suffrage sample, 1921–1937.

Variables	(1)	(2)	(3)	(4)	(5)	(6)
	Current expenditure	Current expenditure	Capital expenditure	Capital expenditure	Administration	Administration
Lagged dep. var.	0.72⁎⁎⁎	0.79⁎⁎⁎	0.45⁎⁎⁎	0.53⁎⁎⁎	0.69⁎⁎⁎	0.78⁎⁎⁎
	[9.93]	[19.4]	[7.71]	[11.7]	[9.37]	[18.3]
Election	− 0.55⁎⁎⁎	− 0.59⁎⁎	0.93⁎⁎	0.94⁎⁎⁎	0.080	0.078
	[− 4.17]	[− 1.97]	[2.35]	[2.72]	[1.44]	[1.23]
Population growth	− 0.000091⁎⁎⁎	− 0.000096⁎⁎	− 0.000047⁎⁎	− 0.000049	− 9.9e − 06⁎⁎	− 0.000012⁎
	[− 3.77]	[− 2.54]	[− 2.22]	[− 1.14]	[− 2.73]	[− 1.70]
Population	− 0.000016⁎⁎	− 0.000018⁎⁎	− 9.0e − 06	− 0.000010	− 1.3e − 06	− 1.9e − 06
	[− 2.18]	[− 1.98]	[− 1.20]	[− 0.97]	[− 0.88]	[− 1.05]
Age structure	− 0.67⁎⁎⁎	− 0.62⁎⁎	0.36	0.30	− 0.13⁎⁎	− 0.13⁎
	[− 2.91]	[− 1.98]	[1.34]	[0.88]	[− 2.45]	[− 1.75]
Population density	− 1.51⁎⁎	− 1.71⁎⁎⁎	− 1.09⁎	− 1.18⁎⁎	− 0.13	− 0.20⁎
	[− 2.20]	[− 3.32]	[− 2.00]	[− 2.04]	[− 1.11]	[− 1.74]
Debt	0.029⁎⁎	0.023⁎⁎⁎	0.047⁎⁎⁎	0.041⁎⁎⁎	0.0038⁎⁎	0.0024⁎⁎
	[2.49]	[3.58]	[3.35]	[5.82]	[2.65]	[2.11]
Wealth	0.0040⁎⁎	0.0039⁎⁎⁎	0.0031⁎⁎⁎	0.0031⁎⁎⁎	0.00046	0.00044⁎⁎⁎
	[2.38]	[7.01]	[4.61]	[4.81]	[1.48]	[4.22]
Estimation method	Fixed effects^a^	LSDV^b^	Fixed effects^a^	LSDV^b^	Fixed effects^a^	LSDV^b^
Observations	448	448	448	448	392^c^	392^c^
R-squared	0.87		0.54		0.81	
Number of boroughs	28	28	28	28	28	28

Note: All fiscal variables are expressed in real Pounds per 1000 capita.

a Robust z-statistics clustered at the borough level in brackets; borough fixed effects included.

b Bias corrected LSDV dynamic panel data estimator suggested by Bruno (2005a,b).

c Sample from 1923 to 1937.

⁎⁎⁎ p < 0.01.

⁎⁎ p < 0.05.

⁎ p < 0.1.

**Table 8:** Robustness checks for the taxpayer suffrage sample, 1902–14.

Variables	(1)	(2)	(3)	(4)	(5)	(6)	(7)
	Current income	Capital income	Rate income	Deficit	Current expenditure	Capital expenditure	Administration
*Panel A*							
Election	− 0.47⁎⁎⁎	− 1.43	− 0.49⁎⁎	0.36⁎⁎⁎	− 0.022	− 1.08	− 0.032⁎⁎⁎
	[− 3.29]	[− 1.26]	[− 2.54]	[2.79]	[− 0.10]	[− 1.04]	[− 4.54]
Left	− 0.16	4.01	0.14	− 0.034	0.42⁎	3.43	− 0.012
	[− 0.52]	[1.56]	[0.25]	[− 0.22]	[1.74]	[1.29]	[− 0.63]

*Panel B*							
Election	− 0.47⁎⁎⁎	− 1.57	− 0.50⁎⁎	0.36⁎⁎	− 0.039	− 1.19	− 0.032⁎⁎⁎
	[− 3.24]	[− 1.30]	[− 2.55]	[2.74]	[− 0.17]	[− 1.07]	[− 4.53]
Absent owners	− 0.000080	0.00037	− 0.00023	− 0.00011	0.00033	0.00029	− 0.000027
	[− 0.15]	[0.30]	[− 0.24]	[− 0.33]	[0.63]	[0.23]	[− 0.65]
Estimation method	Fixed effects^a^	Fixed effects^a^	Fixed effects^a^	Fixed effects^a^	Fixed effects^a^	Fixed effects^a^	Fixed effects^a^
Observations	336	336	336	336	336	336	336
Number of boroughs	28	28	28	28	28	28	28

Note: All fiscal variables are expressed in real Pounds per 1000 capita. All estimations include a lagged dependent variable and the same control variables as in Table 4. The results are similar with the LSDV estimator.

a Robust z-statistics clustered at the borough level in brackets; borough fixed effects included.

⁎⁎⁎ p < 0.01.

⁎⁎ p < 0.05.

⁎ p < 0.1.

**Table 9:** Robustness checks for the universal suffrage sample, 1921–37.

Variables	(1)	(2)	(3)	(4)	(5)	(6)	(7)
	Current income	Capital income	Rate income	Deficit	Current expenditure	Capital expenditure	Administration
*Panel A*							
Election	− 1.02⁎⁎⁎	0.59	− 0.21	0.47⁎⁎⁎	− 0.55⁎⁎⁎	0.93⁎⁎	0.084
	[− 4.43]	[1.31]	[− 0.34]	[3.14]	[− 4.13]	[2.35]	[1.43]
Left	0.72	− 0.23	1.20	0.067	0.74	0.077	− 0.070
	[1.24]	[− 0.40]	[1.40]	[0.33]	[1.18]	[0.099]	[− 0.50]

*Panel B*							
Election	− 1.70⁎⁎⁎	0.57	− 0.87⁎	0.71⁎⁎⁎	− 1.15⁎⁎⁎	0.92⁎⁎	− 0.0093
	[− 6.97]	[1.26]	[− 1.94]	[3.48]	[− 7.46]	[2.37]	[− 0.15]
Great Depression	6.16⁎⁎⁎	0.21	4.95⁎⁎	− 0.76⁎⁎	5.40⁎⁎⁎	0.020	0.60⁎⁎⁎
	[7.82]	[0.48]	[2.14]	[− 2.57]	[9.26]	[0.037]	[6.05]
Estimation method	Fixed effects^a^	Fixed effects^a^	Fixed effects^a^	Fixed effects^a^	Fixed effects^a^	Fixed effects^a^	Fixed effects^a^
Observations	448	448	392	448	448	448	392
Number of boroughs	28	28	28	28	28	28	28

Note: All fiscal variables are expressed in real Pounds per 1000 capita. All estimations include a lagged dependent variable and the same control variables as in Table 5. The results are similar with the LSDV estimator.

⁎⁎⁎ p < 0.01.

⁎⁎ p < 0.05.

⁎ p < 0.1.

a Robust z-statistics clustered at the borough level in brackets; borough fixed effects included.

**Table 10:** The election year effect by borough for the seven fiscal outcomes, 1902–14.

Borough	FE	(1)	(2)	(3)	(4)	(5)	(6)	(7)
		Current income	Capital income	Rate income	Deficit	Current expenditure	Capital expenditure	Administration
Stepney	38	**0.55**⁎⁎⁎	0.48	**0.20**⁎⁎⁎	**0.48**⁎⁎⁎	− 0.38⁎⁎⁎	− 0.023	− 0.024⁎⁎⁎
Hammersmith	50	− 1.66⁎⁎⁎	0.078	− 1.37⁎⁎⁎	**0.32**⁎⁎⁎	**0.48**⁎⁎⁎	− 2.11⁎⁎⁎	− 0.038⁎⁎⁎
Islington	53	–0.42⁎⁎⁎	− 0.77⁎	–0.62⁎⁎⁎	− 0.022	− 0.16⁎⁎⁎	− 0.90⁎⁎	− 0.013⁎⁎⁎
Poplar	53	**0.47**⁎⁎⁎	–0.98⁎⁎⁎	–0.37⁎⁎⁎	**1.29**⁎⁎⁎	**0.45**⁎⁎⁎	− 0.76⁎	− 0.046⁎⁎⁎
Bethnal Green	57	0.00043	− 0.65⁎	–0.31⁎⁎⁎	− 0.29⁎⁎⁎	**0.13**⁎⁎⁎	− 0.32	− 0.015⁎⁎⁎
St. Pancras	57	–0.56⁎⁎⁎	− 0.0044	–0.47⁎⁎⁎	0.16	**0.23**⁎⁎⁎	− 0.59	− 0.036⁎⁎⁎
Greenwich	58	**0.85**⁎⁎⁎	− 0.19	**0.44**⁎⁎⁎	**0.43**⁎⁎⁎	− 0.29⁎⁎⁎	− 0.49	− 0.026⁎⁎⁎
Lambeth	59	–0.87⁎⁎⁎	− 0.59	–0.77⁎⁎⁎	**0.29**⁎⁎	− 0.041	− 0.72	− 0.033⁎⁎⁎
Stoke-Newington	59	**0.36**⁎⁎⁎	− 0.13	–0.35⁎⁎⁎	− 0.46⁎⁎⁎	− 0.24⁎⁎⁎	0.31	− 0.096⁎⁎⁎
Woolwich	59	–1.68⁎⁎⁎	–3.37⁎⁎⁎	–1.52⁎⁎⁎	**0.84**⁎⁎⁎	− 0.53⁎⁎⁎	− 4.09⁎⁎⁎	− 0.097⁎⁎⁎
Southwark	61	–0.81⁎⁎⁎	− 0.12	–0.96⁎⁎⁎	− 0.38⁎⁎⁎	− 0.096	0.14	− 0.022⁎⁎⁎
Deptford	62	–1.01⁎⁎⁎	− 0.61	–0.74⁎⁎⁎	− 0.72⁎⁎⁎	**0.097**⁎⁎⁎	0.036	**0.029**⁎⁎⁎
Hackney	62	–0.65⁎⁎⁎	− 1.17⁎⁎	–0.78⁎⁎⁎	− 0.72⁎⁎⁎	− 0.0014	− 0.25	− 0.057⁎⁎⁎
Westminster	62	–1.12⁎⁎⁎	− 1.92	–2.09⁎⁎⁎	− 0.30⁎⁎⁎	**4.99**⁎⁎⁎	1.21	− 0.12⁎⁎⁎
Bermondsey	63	–1.23⁎⁎⁎	− 2.09⁎⁎⁎	–0.92⁎⁎⁎	**0.51**⁎⁎⁎	− 0.13⁎⁎⁎	− 0.50	− 0.034⁎⁎⁎
Chelsea	63	− 0.24⁎⁎	− 1.73⁎⁎	–1.18⁎⁎⁎	**0.97**⁎⁎⁎	− 0.35⁎⁎⁎	− 0.78	− 0.031⁎⁎⁎
Fulham	63	0.11	–2.71⁎⁎⁎	–0.17⁎⁎⁎	**0.74**⁎⁎⁎	− 0.99⁎⁎⁎	− 1.47⁎⁎⁎	− 0.015⁎⁎⁎
St. Marylebone	63	–0.46⁎⁎⁎	–21.4⁎⁎⁎	**0.65**⁎⁎⁎	**0.50**⁎⁎⁎	0.15	− 17.3⁎⁎⁎	− 0.00018
Finsbury	64	–0.31⁎⁎⁎	− 1.32⁎	–0.95⁎⁎⁎	**0.58**⁎⁎⁎	− 0.082⁎	− 1.18	− 0.041⁎⁎⁎
Shoreditch	64	0.13	–1.58⁎⁎⁎	**2.29**⁎⁎⁎	− 0.56⁎⁎⁎	− 0.26⁎⁎⁎	0.10	− 0.025⁎⁎⁎
Holborn	66	–1.01⁎⁎⁎	− 1.93	–0.69⁎⁎⁎	**1.23**⁎⁎⁎	− 1.30⁎⁎⁎	− 1.12	− 0.032⁎⁎⁎
Camberwell	67	–0.64⁎⁎⁎	0.12	− 0.72⁎⁎⁎	**0.20**⁎⁎⁎	− 0.065⁎	− 0.085	**0.036**⁎⁎⁎
Kensington	67	–0.54⁎⁎⁎	− 1.02	–0.29⁎⁎⁎	**0.85**⁎⁎⁎	**0.85**⁎⁎⁎	− 1.44⁎	0.013⁎⁎⁎
Paddington	68	–0.29⁎⁎	− 0.41	− 0.26⁎⁎	**0.36**⁎⁎⁎	− 0.33⁎⁎⁎	− 0.92	− 0.031⁎⁎⁎
Hampstead	70	–2.15⁎⁎⁎	− 2.01⁎⁎	–1.28⁎⁎⁎	**1.78**⁎⁎⁎	− 0.97⁎⁎⁎	− 1.96⁎⁎⁎	0.00024
Lewisham	70	–0.28⁎⁎⁎	**0.73**⁎	− 0.34⁎⁎⁎	**0.72**⁎⁎⁎	**0.21**⁎⁎⁎	0.061	− 0.081⁎⁎⁎
Wandsworth	70	**0.90**⁎⁎⁎	− 0.89⁎⁎	**0.60**⁎⁎⁎	**0.31**⁎⁎⁎	− 0.099	− 0.41	− 0.049⁎⁎⁎
Battersea	71	–0.67⁎⁎⁎	− 0.040	–1.20⁎⁎⁎	**0.79**⁎⁎⁎	− 0.70⁎⁎⁎	**0.98**⁎⁎	− 0.0077⁎⁎⁎

Note: FE is *franchise extension* (the suffrage as a percentage of the adult male population). The coefficients reported are borough specific election year effects. Significant positive election effects are in bold. All estimations include borough fixed effect and the control variables reported in Tables 4 and 5. Stars are based on robust z-statistics clustered at the borough level.

⁎⁎⁎ p < 0.01.

⁎⁎ p < 0.05.

⁎ p < 0.1.

**Table 11:** The election year effect by borough for the seven fiscal outcomes, 1921–37.

Borough	(1)	(2)	(3)	(4)	(5)	(6)	(7)
	Current income	Capital income	Rate income	Deficit	Current expenditure	Capital expenditure	Administration
Battersea	− 1.82⁎⁎⁎	− 0.16⁎⁎⁎	− 0.87⁎⁎⁎	**1.22**⁎⁎⁎	− 0.50⁎⁎⁎	**0.24**⁎⁎⁎	− 0.16⁎⁎⁎
Bermondsey	**2.20**⁎⁎⁎	− 2.91⁎⁎⁎	**4.10**⁎⁎⁎	− 1.15⁎⁎⁎	− 0.63⁎⁎⁎	− 3.12⁎⁎⁎	**0.065**⁎
Bethnal Green	− 0.95⁎⁎⁎	**2.80**⁎⁎⁎	− 2.55⁎⁎⁎	**0.76**⁎⁎⁎	− 0.51⁎⁎⁎	− 2.70⁎⁎⁎	− 0.19⁎⁎⁎
Camberwell	**0.60**⁎⁎⁎	− 0.11⁎	**1.01**⁎⁎⁎	0.17	0.55⁎⁎⁎	− 0.63⁎⁎⁎	− 0.023⁎
Chelsea	− 0.96⁎⁎⁎	− 2.63⁎⁎⁎	− 0.56⁎⁎⁎	− 0.087	− 1.80⁎⁎⁎	− 1.69⁎⁎⁎	0.0020
Westminster	− 0.78	**4.83**⁎⁎⁎	**17.7**⁎⁎	**1.97**⁎⁎⁎	0.34	**3.41**⁎⁎⁎	0.18
Deptford	− 2.39⁎⁎⁎	**1.55**⁎⁎⁎	− 1.18⁎⁎⁎	**0.43**⁎⁎⁎	− 1.21⁎⁎⁎	**0.41**⁎⁎⁎	− 0.070⁎⁎⁎
Finsbury	− 2.90⁎⁎⁎	**4.34**⁎⁎⁎	− 0.75⁎	**1.88**⁎⁎⁎	− 0.92⁎⁎⁎	**7.18**⁎⁎⁎	− 0.12⁎⁎⁎
Fulham	− 1.11⁎⁎⁎	− 1.44⁎⁎⁎	0.052	**0.76**⁎⁎⁎	0.0063	0.13	**0.11**⁎⁎⁎
Greenwich	− 1.18⁎⁎⁎	**5.02**⁎⁎⁎	− 0.30⁎⁎	**0.48**⁎⁎⁎	− 0.95⁎⁎⁎	**3.01**⁎⁎⁎	**0.27**⁎⁎⁎
Hackney	− 1.60⁎⁎⁎	**1.30**⁎⁎⁎	− 1.04⁎⁎⁎	**0.68**⁎⁎⁎	− 0.77⁎⁎⁎	**0.11**⁎	− 0.062⁎⁎⁎
Hammersmith	− 0.88⁎⁎⁎	− 3.84⁎⁎⁎	− 0.22	0.37⁎⁎	− 0.86⁎⁎⁎	**1.59**⁎⁎⁎	**1.06**⁎⁎⁎
Hampstead	0.55⁎⁎⁎	− 1.08⁎⁎⁎	− 0.33⁎⁎⁎	− 0.74⁎⁎⁎	− 0.56⁎⁎⁎	**2.34**⁎⁎⁎	**0.24**⁎⁎⁎
Holborn	− 1.97⁎⁎⁎	0.10	− 6.91⁎⁎⁎	**2.02**⁎⁎⁎	− 1.96⁎⁎⁎	**0.94**⁎⁎⁎	− 0.37⁎⁎⁎
Islington	− 2.70⁎⁎⁎	**1.47**⁎⁎⁎	− 0.80	**1.63**⁎⁎⁎	− 0.85⁎⁎⁎	**1.44**⁎⁎⁎	0.048⁎
Kensington	− 0.66⁎⁎⁎	− 1.74⁎⁎⁎	− 0.18	**0.73**⁎⁎⁎	− 0.18⁎⁎⁎	**0.26**⁎⁎⁎	− 0.036⁎⁎
Lambeth	− 1.36⁎⁎⁎	**1.09**⁎⁎⁎	− 0.50⁎⁎⁎	**0.51**⁎⁎⁎	− 0.76⁎⁎⁎	− 0.44⁎⁎⁎	− 0.0035
Lewisham	− 0.40⁎⁎⁎	**2.08**⁎⁎⁎	0.36⁎	0.072	− 0.83⁎⁎⁎	**0.72**⁎⁎⁎	− 0.024⁎
Paddington	**0.91**⁎⁎⁎	**1.36**⁎⁎⁎	0.95⁎⁎⁎	− 0.078	− 0.47⁎⁎⁎	**0.57**⁎⁎⁎	**0.032**⁎⁎
Poplar	**0.59**⁎⁎⁎	**− 1.22**⁎⁎⁎	**1.86**⁎⁎⁎	− 0.24	− 0.69⁎⁎⁎	− 0.85⁎⁎⁎	− 0.24⁎⁎⁎
Shoreditch	− 1.37⁎⁎⁎	− 1.68⁎⁎⁎	− 0.84⁎⁎⁎	0.027	− 0.98⁎⁎⁎	− 1.26⁎⁎⁎	**0.26**⁎⁎⁎
Southwark	− 3.35⁎⁎⁎	− 0.32⁎⁎⁎	− 1.69⁎⁎⁎	**1.73**⁎⁎⁎	− 1.11⁎⁎⁎	− 0.35⁎⁎⁎	**0.56**⁎⁎⁎
St. Marylebone	− 2.43⁎⁎⁎	**2.92**⁎⁎⁎	− 3.58⁎⁎⁎	**1.03**⁎⁎⁎	− 0.88⁎⁎⁎	**3.96**⁎⁎⁎	**0.091**⁎⁎⁎
St. Pancras	− 0.41⁎⁎⁎	**1.82**⁎⁎⁎	− 0.69⁎⁎⁎	0.19	− 0.025	**1.59**⁎⁎⁎	− 0.044⁎⁎
Stepney	− 1.80⁎⁎⁎	− 1.10⁎⁎⁎	− 2.67⁎⁎⁎	**0.68**⁎⁎⁎	− 0.36	**1.86**⁎⁎⁎	**0.082**⁎⁎⁎
Stoke − Newington	− 1.09⁎⁎⁎	**2.84**⁎⁎⁎	− 2.02⁎⁎⁎	**1.81**⁎⁎⁎	**1.31**⁎⁎⁎	**1.81**⁎⁎⁎	**0.037**⁎⁎⁎
Wandsworth	− 0.44⁎⁎⁎	**2.66**⁎⁎⁎	− 0.55⁎⁎⁎	0.16	− 0.20⁎⁎⁎	**1.83**⁎⁎⁎	**0.49**⁎⁎⁎
Woolwich	− 0.86⁎⁎⁎	− 1.31⁎⁎⁎	− 0.76⁎⁎⁎	**0.93**⁎⁎⁎	**0.60**⁎⁎⁎	**3.68**⁎⁎⁎	**0.054**⁎⁎⁎

Note: The coefficients reported are borough specific election year effects. Significant positive election effects are in bold. All estimations include borough fixed effect and the control variables reported in Tables 6 and 7. Stars are based on robust z-statistics clustered at the borough level.

⁎⁎⁎ p < 0.01.

⁎⁎ p < 0.05.

⁎ p < 0.1.

## Footnotes

2 See, e.g., Aidt and Jensen (2009) for evidence that the likelihood of (progressive) income tax during the long 19th century is reduced by suffrage reform; and Kenny and Winer (2006) for evidence from the post-war period that greater political freedom does not necessarily lead to more redistribution. Aidt et al. (2010) show that franchise reform can lead to a reduction in spending. See also Lindert (1994, 2004a,b), Husted and Kenny (1997), Aidt et al. (2006), Aidt and Eterovic (2011), Aidt and Jensen (2013), and Engerman and Sokoloff (2011).

3 See, for example, Paldam (1979), Roubini and Sachs (1989), Alesina et al. (1992), Blais and Nadeau (1992), Rosenberg (1992), Schuknecht (1996), Franzese (2000), Seitz (2000), Kneebone and McKenzie (2001), Galli and Rossi (2002), Akhmedov and Zhuravskaya (2004), Andrikopoulos et al. (2004), Shi and Svensson (2006), Veiga and Veiga (2007), Baleiras and Costa (2004), Mink and de Haan (2006), Foucault et al. (2008), Brender and Drazen (2008), Vergne (2009), Drazen and Eslava (2010), Potrafke (2010, 2012), Efthyvoulou (2011), Sakurai and Menezes-Filho (2011), Aidt et al. (2011), and Klomp and De Haan (2013b). The literature was initiated by the classical papers by Nordhaus (1975) and Rogoff and Sibert (1988).

4 While the franchise rules may also affect the nature of party politics, we do not expect systematically different partisan cycles (budget cycles which are driven by differences in the ideological preposition of the majority party) under the two regimes. We use a robustness check to test the validity of this assumption.

5 The suffrage was neither equal (in that, for example, graduates of certain universities could vote twice) nor universal (in that it excluded unmarried and younger women who had to wait until 1928 to get the vote).

6 Klomp and De Haan (2013a) have recently challenged whether it is appropriate to restrict the coefficient on the election cycle indicator to be homogenous across countries. As with other studies that make use of local government data, e.g., Veiga and Veiga (2007), our study is less open to this critique because the institutional and economic environment is broadly the same across the LMBs.

7 Heckelman and Whaples (1996) is the only other study we are aware of which investigates the political business cycle (in GDP in the USA) from a historical perspective.

8 For a discussion of under-investment in the urban amenities and infrastructure during the 19th century, see Williamson (1990). The life expectancy data reported in Szreter and Mooney (1998) demonstrate that the situation did not improve much until late in the century.

9 We exclude from the analysis the City of London, which was a Corporation and governed by a different set of rules and had access to a particularly large tax base.

10 See Davis (1988, Appendix 4), for the composition of the LMBs.

11 Schools were run by the London School Board (which was abolished in 1904 when the London County Council assumed responsibility for education), law enforcement was run by the Metropolitan Police, and London-wide infrastructure projects fell under the jurisdiction of London County Council, established in 1888.

12 We have only been able to detect changes to this arrangement in the collection of the poor law rate. In March 1921, the Labour-run council in the east end borough of Poplar refused to pay precepts to the central London agencies in an attempt to force equalization of the rates across the capital. Supported by the adjoining boroughs of Bethnal Green and Stepney, 30 Poplar councilors were jailed for their dissent (Booth, 2009). The outcome failed to equalize the rates, but instead pooled more outdoor relief through the Common Poor Fund. Just before the War, 63% of metropolitan guardians' expenditure came through the Common Poor Fund, a figure which rose to 85% following the east end protests (Gillespie, 1989, p. 180). When the Poor Law was abolished, all of its functions were transferred to the London County Council's Public Assistance Committee in 1929 (Gillespie, 1989, p. 182). We stress that these changes did not influence the core operations of the LMBs.

13 See Doyle (2000) for a discussion of the local government organization in England and Wales.

14 This system was common to all elected local government bodies in England and Wales throughout the 19th century which makes it impossible to study the political budget cycle using British local government data before the establishment of the LMBs.

15 These percentages are calculated by dividing the number of eligible voters (taken from London Statistics 1909–10, vol. XX, pp. 24–30) by the male population over the age of 20 (taken from the 1911 census).

16 This is analogous to the assumption in Lohmann (1998) that voters do not observe monetary policy (and inflation) until after the election. Alternatively, we could, as do Shi and Svensson (2006), assume that voters observe some budget components but not all of them.

17 The online supplementary appendix provides all proofs.

18 It is, however, often possible to circumvent budget rules, see, e.g., Rose (2006) or Veiga and Veiga (2007).

19 A lagged dependent variable is included in the estimations, which means that one year of observations is lost and the respective sample sizes used in the regressions are 336 and 448. The detailed accounts for the fiscal year ending 1922 were only published in abbreviated format and some of the disaggregated data is missing for 1922. For these items, the universal suffrage sample is further reduced by two years and contains 392 observations. Abbreviated accounts were published in 1922 because the Ministry of Health was short-staffed.

20 The relevant census years are 1901, 1911, 1921, and 1931. The planned census of 1941 was not carried out because of the Second World War. We interpolated linearly between the census dates.

21 The data on rateable values are recorded in the Local Taxation Returns (1901–1914) and in the Local Government Financial Statistics (1918–1938). The nominal data are deflated by the Sauerbeck-Statisk price index. From Table 3, we note a fall in real rateable value per 1000 houses after the War. The fall can be attributed to two factors. Firstly, nominal valuations did not increase at the same speed as the price index in the early interwar years. Secondly, the working class houses built after the War were valued below average, if at all.

22 The wages of direct-labour employees were a particularly contentious topic in metropolitan politics. Gillespie (1989, p. 170) notes that “Labour-controlled councils awarded minimum wages considerably in excess of trade union rates”. Such policies became a target for the Municipal Reformers, who sought to reduce these sorts of expenditures and keep a firm lid on the rates.

23 Since elections take place at the same time in all boroughs, we cannot include time fixed effects. For a rare study of local elections where the election year effect can be separated from common time effects, see Bambang et al. (2013).

24 Judson and Owen (1999) show that the bias is negligible for panels that cover more than 20 years.

25 We draw on data on the number of seats won by each registered party in each LMB election reported in Willis et al. (2000). We code *left* based on an analysis of seat shares.

26 Brender and Drazen (2005) make a distinction between “new” and “old” democracies and make use of the Polity IV database to do so (Marshall and Jaggers, 2000). The Policy IV index categorises countries according to a range of political authority patterns to arrive at a country score. The index, however, does not capture variations in the suffrage rules directly and so does not effectively distinguish regimes along this dimension.

27 See, e.g., Brender and Drazen (2005), Shi and Svensson (2006) and Alt and Lassen (2006a,b).
