# Supplementary material for: Voting suffrage and the political budget cycle: Evidence from the London Metropolitan Boroughs 1902–1937
Source: J Public Econ. 2014 Apr;112:53–71. doi: 10.1016/j.jpubeco.2014.01.003 (PMC4375611; doi:10.1016/j.jpubeco.2014.01.003)
Supplement: Supplementary file 1 — Supplementary material. [file mmc1.pdf]

Supplementary Online Appendix

(Not Intended for Publication)
to
Voting suffrage and the political budget cycle: evidence from the London Metropolitan Boroughs 1902-1937
by

Toke S. Aidt
University of Cambridge, UK
Graham Mooney
Johns Hopkins University, USA

1 Overview

We develop a moral hazard model of electoral competition in the tradition of Lohmann (1998) and Shi and Svensson (2006) to show how and why the nature of the opportunistic political budget cycle depends on the suffrage rules. The economic and fiscal structure of the model is based on Aidt et al. (2010). The main text sketches the structure and the main results of the model. This appendix fills in the details and provides proofs of the main results.

2 The Economic Structure

The production technology is $y_t = A_t l_t^{\alpha} k^{1-\alpha}, 0 < \alpha < 1$. $A(.)$ represents total factor productivity and l_t is labour demand. Output is sold in the national market at the constant price p. The demand for labour from each capitalist $\ell = k(\frac{\alpha A(.)}{w})^{\frac{1}{1-\alpha}}$. The market clearing wage is $w^*(.) = A(.)z$, where $z = \alpha(\frac{n_C k}{n_L})^{1-\alpha}$. Profits earned by each capitalist are $\pi^*(.) = A(.)kx$, where $x = (1-\alpha)(\frac{n_L}{n_C k})^{\alpha}$. We assume that $A = A(g)$ with $A' > 0$ and $A'' < 0$.

3 The equilibrium budget without elections

The capitalist-politician's balanced budget problem in the absence of elections is

$$\max u_C(p, q, \pi^*(g) - \frac{g+q+r}{n_C} + r) + M \tag{1}$$

subject to $r \leq r^*$. The first order conditions are:

$$\left.\frac{\partial u_C}{\partial m}\right|_{m^P} \left(\frac{\partial \pi^*}{\partial g} - \frac{1}{n_C}\right) = 0 \tag{2}$$

$$\left.\frac{\partial u_C}{\partial q}\right|_{m^P} - \left.\frac{\partial u_C}{\partial m}\right|_{m^P} \frac{1}{n_C} = 0 \tag{3}$$

$$\left.\frac{\partial u_C}{\partial m}\right|_{m^P} \left(1 - \frac{1}{n_C}\right) \geq 0, \tag{4}$$

where we have made it explicit that the derivatives of the indirect utility functions are evaluated at the income of the politician (m^P) which is larger than the income of a capitalist in general (m^C) because the politician extracts rents. Equation (2) has a unique solution for g which we call g^*. This is independent of the income level. Equation (4) implies a corner solution with $r = r^*$. This is because the tax cost of rents is shared amongst all capitalists. Given $\{r^*, g^*\}$, we can solve equation (3) for $q^* = q(r^*, g^*)$. A maximum requires that

$$\Phi \equiv \frac{\partial^2 u_c}{\partial q^2} + \left(\frac{1}{n_C}\right)^2 \frac{\partial^2 u_c}{\partial m^2} - 2\frac{\partial^2 u_c}{\partial q \partial m}\frac{1}{n_C} < 0, \tag{5}$$

where all derivatives are evaluated at m^P. The marginal utility of the non-productive public good q and income are both declining. The cross derivative $\frac{\partial^2 u_c}{\partial q \partial m}$ controls if good q is valued more or less at the margin as income increases. If it is a normal good, then $\frac{\partial^2 u_c}{\partial q \partial m} > 0$ and $\Phi < 0$ for sure. If good q is an inferior good, then $\frac{\partial^2 u_c}{\partial q \partial m} < 0$ and we require that this is not "too" negative to insure that $\Phi < 0$. For simplicity, we focus on the case where $\frac{\partial^2 u_c}{\partial q \partial m}$ is positive but note that all results go through for the case where $\frac{\partial^2 u_c}{\partial q \partial m}$ is negative as long as $\Phi < 0$ and

$$\frac{\partial^2 u_c}{\partial q \partial m} - \frac{1}{n_C}\frac{\partial^2 u_c}{\partial m^2} > 0. \tag{6}$$

We use the Implicit Function Theorem to find the derivatives of $q^* = q(r, g)$:

$$\frac{dq^*}{dr} = -\frac{\left(1 - \frac{1}{n_C}\right)\left(\frac{\partial^2 u_c}{\partial q \partial m} - \frac{1}{n_C}\frac{\partial^2 u_c}{\partial m^2}\right)}{\Phi} > 0 \tag{7}$$

$$\frac{dq^*}{dg} = -\frac{\left(\frac{\partial \pi^*}{\partial g} - \frac{1}{n_c}\right)\left(\frac{\partial^2 u_c}{\partial q \partial m} - \frac{1}{n_C}\frac{\partial^2 u_c}{\partial m^2}\right)}{\Phi}.$$

We note that $\frac{dq^*}{dg}$ is negative for $g > g^*$ because $\left.\frac{\partial \pi^*}{\partial g}\right|_{g > g^*} - \frac{1}{n_c} < 0$. The optimal budget in the absence of elections is $\{g^*, q^*, r^*\}$ and the tax rate is $\tau^* = \frac{g^* + q^* + r^*}{2n_C}$. This is the equilibrium post-election budget.

It is useful to compare this solution to the optimal budget allocation as seen from the point of view of a capitalist-voter. The only difference between a capitalist-politician and a capitalist-voter is that the former benefits from rent and, therefore, has higher income. Since the marginal benefit of the productive public good is independent of income, all capitalists want the same level of g (namely, g^*). Capitalist-voters want rents cut to zero and since they have (weakly) lower income than the capitalist-politician, they want less spending on the non-productive public good (q).

4 The utility targets

In period 2, the maximum budget-related utility for the capitalist-politician is $S_C^* = S_C(g^*, q^*, r^*)$. The overall welfare of residents in the two groups are

$$V_2^{C*} = v_C(g^*, q^*) + \eta_2' + \mu_2 \quad (8)$$

$$V_2^{L*} = v_L(g^*) + \eta_2' + \mu_2 \quad (9)$$

where

$$\eta_2' = \rho\eta_1 + (1-\rho)\,\eta_2 \quad (10)$$

and ρ is an indicator function for whether the incumbent from period 1 is re-elected or not. Since all capitalist-politicians implement the same post-election budget, the only reason voters care about who gets elected is that quality varies. As seen from period 1, the expected quality of the capitalist-politician elected for period 2 is

$$E_1\eta_2' = \Pr(\rho=1)E_1\eta_1 + (1-\Pr(\rho=1))\,E_1(E_2\eta_2) = \Pr(\rho=1)E_1\eta_1 \quad (11)$$

since the expected quality of a new capitalist-politician is zero on average ($E_1(E_2\eta_2) = E_2\eta_2 = 0$). The pivotal voters, whether capitalists or workers, want to re-elect the incumbent if and only their estimate at the end of period 1 of the quality of the capitalist-politician who served them during period 1 is positive. That is, if and only if $E_1\eta_1 > 0$. To form a rational (Bayesian) estimate of the expected quality of the incumbent, the pivotal voters use information on observed total utility V_1^i and their knowledge about the equilibrium budget strategy of the incumbent. The equilibrium budget strategy of the incumbent generates a budget-related utility outcome which we call v_{i1}^S. Recall that the total utility of residents in group i is $V_1^i = v_{i1} + \eta_1 + \mu_1$. We can rewrite this by subtracting the equilibrium budget-related utility v_{i1}^S on both sides of the equation

$$\begin{aligned} V_1^i - v_{i1}^S &= v_{i1} + \eta_1 + \mu_1 - v_{i1}^S \quad (12) \\ &= \eta_1 + \mu_1, \end{aligned}$$

where we get the last line by making use of the fact that at equilibrium $v_{i1}^S = v_{i1}$. This means that using their knowledge of the equilibrium, voters can infer the sum of the two shocks. A rational voter can then solve the resulting signal extraction problem and estimate that

$$E_1\eta_1 = \frac{\sigma_\eta^2}{\sigma_\eta^2 + \sigma_\mu^2}\left(V_{i1} - v_{i1}^S\right). \quad (13)$$

From this, it follows that the incumbent capitalist-politician will be re-elected if realized total utility is larger than the budget-related utility voters expect the incumbent to deliver in equilibrium (v_{i1}^S), i.e., if

$$V_{i1} - v_{i1}^S = \eta_1 + \mu_1 + v_{i1} - v_{i1}^S > 0. \quad (14)$$

We can rewrite this as

$$\eta_1 + \mu_1 > -v_{i1} + v_{i1}^S. \tag{15}$$

Let the distribution function for $\eta_1 + \mu_1$ be F and the density function be f. Given the rational retrospective voting strategy – "if $\left(V_{i1} - v_{i1}^S\right) > 0$, then re-elect, otherwise elect a new capitalist-politician" – the probability of re-election, as perceived by the incumbent, is

$$\Pr(\rho = 1) = 1 - F\left(-v_{i1} + v_{i1}^S\right). \tag{16}$$

This re-election probability is increasing in the actual budget-related utility that the incumbent's budget choice generates. This provides an incentive to adjust the budget to please the pivotal voters. To find the equilibrium value of v_{i1}, we write the period 1 utility of the incumbent politician directly as a function of the budget-related utility he provides to the pivotal voters, $S_C(v_{i1})$. The incumbent capitalist-politician increases his re-election chance by providing higher welfare to the pivotal voters, but since this is costly to do, he carefully balances the costs and benefits. The equilibrium value of v_{i1} is that which maximizes the incumbent's inter-temporal payoff, i.e.,

$$\max_{v_{it}} S_C(v_{i1}) + M + \left(1 - F\left(-v_{i1} + v_{i1}^S\right)\right)(S_C^* + M),$$

where we ignore discounting. The first order condition evaluated at equilibrium ($v_{i1}^S = v_{i1}$) is

$$\frac{\partial S_C}{\partial v_{i1}} + f(0)(S_C^* + M) \leq 0. \tag{17}$$

This first order condition has an interior solution under both suffrage regimes. To see this, start from the balanced budget policy $\{g^*, q^*, r^*\}$. A small permutation designed to increase voters' welfare induces a second order reduction in S_C for period 1 but it gives a first order benefit in terms of expected benefit for period 2. This establishes that voters get higher budget-related welfare in period 1 than in period 2. We call the resulting "utility targets" for U_{TS} and U_{US} and note that $U_{TS} > v_C(g^*, q^*, r^*)$ and $U_{US} > v_L(g^*)$.

5 The pre-election equilibrium budgets

Under taxpayer suffrage, the equilibrium balanced budget for period 1 is the solution to (we omitted time subscripts for simplicity):

$$\max S_C(g, q, r) \tag{18}$$

subject to

$$v_C(g, q, r) \geq U_{TS} \tag{19}$$
$$r \leq r^*. \tag{20}$$

The Lagrangian function is

$$L = S_C(g, q, r) + \lambda_1(v_C(g, q, r) - U_{TS}) + \lambda_2(r - r^*) \tag{21}$$

where we recall that

$$S_C(g, q, r) = u_C(p, q, \pi^*(g) - \frac{g + q + r}{n_C} + r) \tag{22}$$

and

$$v_C(g, q, r) = u_C(p, q, \pi^*(g) - \frac{g + q + r}{n_C}). \tag{23}$$

λ_1 and λ_2 are the Lagrange multipliers on the two constraints. The Kuhn-Tucker conditions are

$$\frac{\partial L}{\partial g} = \left(\frac{\partial u_C}{\partial m}\bigg|_{m^C} + \lambda_1 \frac{\partial u_C}{\partial m}\bigg|_{m^P} \right) \left(\frac{\partial \pi^*}{\partial g} - \frac{1}{n_C} \right) = 0 \tag{24}$$

$$\frac{\partial L}{\partial q} = \left(\frac{\partial u_C}{\partial q}\bigg|_{m^P} - \frac{\partial u_C}{\partial m}\bigg|_{m^P} \frac{1}{n_C} \right) + \lambda_1 \left(\frac{\partial u_C}{\partial q}\bigg|_{m^C} - \frac{\partial u_C}{\partial m}\bigg|_{m^C} \frac{1}{n_C} \right) = 0 \tag{25}$$

$$\frac{\partial L}{\partial r} = \frac{\partial u_C}{\partial m}\bigg|_{m^P} \left(1 - \frac{1}{n_C} \right) - \lambda_1 \frac{\partial u_C}{\partial m}\bigg|_{m^C} \frac{1}{n_C} + \lambda_2 \geq 0 \tag{26}$$

along with the two constraints. The re-election constraint binds so the solution must satisfy

$$u_C(p, q, \pi(g) - \frac{g + q + r}{n_C}) = U_{TS} \tag{27}$$

and $\lambda_1 > 0$. Moreover, condition (24) implies that $g_{TS} = g^*$, i.e., there is no pre-election distortion in the delivery of productive public goods. We assume that $u_C(p, q_C(g^*, r^*), \pi(g^*) - \frac{g^* + q_C(g^*, r^*) + r^*}{n_C}) < U_{TS}$. This implies that the re-election constraint cannot be met by cutting q down to the level preferred by capitalist-voters while keeping the rent at the maximum. Accordingly, $\lambda_2 = 0$ as rents are below the maximum ($r_{TS} < r^*$). Conditions (25) and (26) must hold with equality. Since capitalist-voters, for given r and g, want less q than the capitalist-politician, condition (25) implies that $q_{TS} < q^*$. The tax rate is, therefore, lower than τ^*. In short, $g_{TS} = g^*$, $r_{TS} < r^*$, $\tau_{TS} < \tau^*$ and $q_{TS} < q^*$.

Under universal suffrage, the equilibrium pre-election balanced budget is the solution to

$$\max S_C(g, q, r) \tag{28}$$

subject to

$$v_L(g) \geq U_{US} \tag{29}$$

$$r \leq r^*. \tag{30}$$

The re-election constraint binds so the solution must involve $g_{US} = v_L^{-1}(U_{US})$. This is unique because wages are monotonically increasing in g. Clearly, $g_{US} > g^*$. Given this, q and r are chosen to maximize $S_C(g_{US}, q, r)$ subject $r \leq r^*$.

The first order conditions (evaluated at g_{US} and at the income of the capitalist-politician) are

$$\left.\frac{\partial u_C}{\partial q}\right|_{m^P, g_{US}} - \left.\frac{\partial u_C}{\partial m}\right|_{m_P, g_{US}} \frac{1}{n_C} = 0 \tag{31}$$

$$\left.\frac{\partial u_C}{\partial m}\right|_{m^P, g_{US}} \left(1 - \frac{1}{n_C}\right) \geq 0 \tag{32}$$

Clearly, $r_{US} = r^*$. The solution to condition (31) is $q_{US} = q(r^*, g_{US})$. Recall that $\frac{\partial q}{\partial g} < 0$ for $g_{US} > g^*$. It follows that $q_{US} < q^*$. The tax rate is $\tau_{US} = \frac{g_{US}+q_{US}+r^*}{2n_C}$. This may be larger or smaller than τ^*.

These results are derived under the assumption that the full incidence of the property tax falls on capitalists and that the politician is always a capitalist. Both assumptions can be relaxed. In particular, the results are unaffected if most of the incidence (but not all) falls on owners and we can allow workers to run the council as long as the council's objective function under universal suffrage puts some weight on the utility of capitalists.
